# *Spiroplasma* endosymbiont reduction of host lipid synthesis and Stomoxyn-like peptide contribute to trypanosome resistance in the tsetse fly *Glossina fuscipes*

**DOI:** 10.1371/journal.ppat.1012692

**Published:** 2025-01-31

**Authors:** Erick O. Awuoche, Gretchen Smallenberger, Daniel L. Bruzzese, Alessandra Orfano, Brian L. Weiss, Serap Aksoy

**Affiliations:** Department of Epidemiology of Microbial Diseases, Yale School of Public Health, New Haven, Connecticut, United States of America; Heidelberg University, GERMANY

## Abstract

Tsetse flies (*Glossina* spp.) vector African trypanosomes that cause devastating diseases in humans and domestic animals. Within the *Glossina* genus, species in the *Palpalis* subgroup exhibit greater resistance to trypanosome infections compared to those in the *Morsitans* subgroup. Varying microbiota composition and species-specific genetic traits can significantly influence the efficiency of parasite transmission. Notably, infections with the endosymbiotic bacterium *Spiroplasma* have been documented in several *Palpalis* subgroup species, including *Glossina fuscipes fuscipes* (*Gff*). While *Spiroplasma* infections in *Gff* are known to hinder trypanosome transmission, the underlying mechanisms remain unknown. To investigate *Spiroplasma-*mediated factors affecting *Gff* vector competence, we conducted high-throughput RNA sequencing of the gut tissue along with functional assays. Our findings reveal elevated oxidative stress in the gut environment in the presence of *Spiroplasma*, evidenced by increased expression of *nitric oxide synthase*, which catalyzes the production of trypanocidal nitric oxide. Additionally, we observed impaired lipid biosynthesis leading to a reduction of this important class of nutrients essential for parasite and host physiologies. In contrast, trypanosome infections in *Gff’s* midgut significantly upregulated various immunity-related genes, including a small peptide, *Stomoxyn-like*, homologous to *Stomoxyn* first discovered in the stable fly, *Stomoxys calcitrans*. We observed that the *Stomoxyn-like* locus is exclusive to the genomes of *Palpalis* subgroup tsetse species. *GffStomoxyn* is constitutively expressed in the cardia (proventriculus) and synthetic *Gff*Stomoxyn exhibits potent activity against *Escherichia coli* and bloodstream form of *Trypanosoma brucei* parasites, while showing no effect against insect stage procyclic forms or tsetse’s commensal endosymbiont *Sodalis in vitro*. Reducing *Gff*Stomoxyn levels significantly increased trypanosome infection prevalence, indicating its potential trypanocidal role *in vivo*. Collectively, our results suggest that the enhanced resistance to trypanosomes observed in *Spiroplasma*-infected *Gff* may be due to the reduced lipid availability necessary for parasite metabolic maintenance. Furthermore, *Gff*Stomoxyn could play a crucial role in the initial immune response(s) against mammalian parasites early in the infection process in the gut and prevent gut colonization. We discuss the molecular characteristics of *Gff*Stomoxyn, its spatial and temporal expression regulation and its microbicidal activity against *Trypanosome* parasites. Our findings reinforce the nutritional influences of microbiota on host physiology and host-pathogen dynamics.

## Author summary

The tsetse fly, *Glossina fuscipes fuscipes* (*Gff*) is a prominent vector of African trypanosomes, parasites that cause sleeping sickness disease and is of high public health relevance in the Sab-Saharan Africa. *Gff* exhibits strong innate resistance to trypanosomes, especially when infected with the endosymbiotic bacterium, *Spiroplasma*. This study investigated the mechanisms that underlie *Spiroplasma* mediated resistance to trypanosome infection in *Gff*. Our results indicate alterations in host lipid metabolism with reduced levels of triglycerides, suggesting a potential metabolic barrier that limits the availability to parasite. In addition, we discovered a small peptide, Stomoxyn, exclusively expressed in *Gff* and related *Palpalis* tsetse species. Synthetic *Gff* Stomoxyn exhibits antibacterial and antitrypanosomal properties, and experimentally reducing *Gff stomoxyn* levels increases parasite infection prevalence in the fly. We suggest that strategies to increase *Spiroplasma* prevalence or enhance stomoxyn expression through paratransgenic approaches could be promising avenues for reducing trypanosomiasis transmission.

## Introduction

Tsetse flies (*Glossina* spp.) transmit African trypanosome parasites that cause sleeping sickness (Human African Trypanosomiasis, HAT) in humans and Nagana (Animal African Trypanosomiasis, AAT) in livestock [1]. Approximately 60 million people live in tsetse fly-infested areas in sub-Saharan Africa and hence are at risk of contracting HAT, while AAT is rampant and results in significant loss of agricultural productivity among the farming communities in impoverished areas of the continent [2, 3]. No vaccines exist to prevent mammalian infections due to a process of antigenic variation by which the parasites sequentially express antigenically distinct surface coat proteins to evade vertebrate host immune responses [4]. Reduction of tsetse fly populations can be effective at curbing the disease, but both the challenges and cost of implementing vector control activities and re-infestation risk once the programs are abandoned reduce their efficacy [5]. Blocking or reducing the ability of parasite transmission through the fly has been entertained as an additional method to boost disease control efforts [6–8]. For successful development of such alternative biological methods, better knowledge is required on parasite-vector dynamics, parasite transmission biology and antiparasitic molecules that could interfere with parasite transmission through tsetse fly vector.

Tsetse flies exhibit innate resistance to infection with trypanosomes, with low infection prevalences reported in wild and experimentally colonized fly populations [9–12]. Various factors influence parasite transmission efficiency under experimental conditions, including fly age and nutritional status at time of exposure to the parasite, species/strain of the trypanosome studied and resident microbiota in the fly midgut. Among the four subgroups of *Glossina*—*Fusca*, *Palpalis*, *Morsitans* and *Machadomia*- species within the *Morsitans* subgroup, including the well studied *Glossina morsitans morsitans* (*Gmm*), typically exhibit greater susceptibility to trypanosome infections compared to those in the *Palpalis* subgroup (e.g., *Glossina fuscipes fuscipes (Gff*), *Glossina tachinoides* (*Gt*), *Glossina palpalis palpalis* (*Gpp*) and *Glossina palpalis gambiensis (Gpg)*) [11, 13–15]. The *Palpalis* subgroup, also known as riverine group, is widely distributed in West and Central Africa, covering an estimated area of 6415 square kilometers and living in close association with human habitats [16, 17]. Fly species in this subgroup are highly relevant to public health as they serve as the primary vectors for trypanosomes responsible for chronic sleeping sickness in the regions they inhabit [18, 19].

In addition to ecological differences and host preferences, variations in vector competence between *Morsitans* and *Palpalis* subgroup flies may arise from species-specific genetic content, as evidenced by comparisons of whole-genome sequencing (WGS) data across different *Glossina* subgroups [20]. A comparative analysis of orthology groups (OGs) among the four subgroups revealed the presence of 2223 OGs specific to the *Palpalis* subgroup, with 4948 genes shared between *Gff* and *Gpp* [20]. Notably the *Palpalis* subgroup exhibited gene expansions, including those encoding helicases involved in the production of small RNAs that mediate post-transcriptional gene expression and defensive responses against viruses and transposable elements, alongside gene duplications such as the trypanocidal peptide Cecropins in *Gff* [20, 21]. Laboratory studies in *Gmm* have shown that when flies acquire the bloodstream form (BSF) trypanosomes as newly eclosed adults (teneral) in their first bloodmeal, they exhibit higher susceptibility to parasite infections. However, older adults display greater midgut resistance and can eliminate the BSF trypanosomes early in the infection process in the midgut before they can colonize this organ. The trypanocidal factors described in *Gmm* include the gut peritrophic matrix [22–24], antimicrobial peptides [25–28], reactive oxygen species (ROS) [29, 30] tsetse EP proteins [31], trypanolysin [32–34], peptidoglycan recognition protein (PGRP)-LB [35], lectins [36–38] and other proteolytic enzymes [39–41]. Beyond physical barriers and innate immune factors, gut endosymbionts have also been implicated to influence parasite transmission success. Tsetse flies harbor a species-specific combination of four well-characterized microbes, including *Wigglesworthia*, *Sodalis*, *Wolbachia* and *Spiroplasma*. Each of these endosymbionts display a different evolutionary history with their vector species and exert varying influences on fly physiology. Several investigations have described a positive correlation between trypanosome infection prevalence and the presence of the commensal symbiont *Sodalis*, although the mechanism remains unconfirmed [10, 41]. The mutualist *Wigglesworthia* has been shown to induce the expression of a host amidase with trypanolytic activity (PGRP-LB) that reduces parasite colonization success as an early response during the infection process in the midgut [35].

Infections with the endosymbiont *Spiroplasma glossinidia* (*Spiroplasma*) were reported uniquely from the species within the *Palpalis* subgroup, including *Gff* [42], *Gt*, and *Gpp* [43, 44]. In Uganda, *Spiroplasma* infections have been found to range in prevalence (5–34%) in distinct *Gff* populations in the Northwest region of the country and infections persist stably over time and space with seasonality being one important factor in infection prevalences [45]. Our studies with a *Gff* laboratory line also showed that approximately 50% of adults are infected with S*piroplasma* [46]. Our studies using this *Gff* line suggest that maternal transmission of *Spiroplasma* occurs with high fidelity from infected mothers to each of their offspring. However, we also observed evidence of paternal transmission of *Spiroplasma*, which could explain the heterogenous infections observed in the *Gff* laboratory line [46]. In both field captured and laboratory reared *Gff*, the bacterium resides in reproductive and digestive tissues as well as in hemolymph [42, 47]. *Spiroplasma* infection significantly alters gene expression in reproductive tissues of both male and female *Gff* [46]. We also observed reduced circulating TAG levels in the hemolymph of *Spiroplasma*-infected pregnant females accompanied by an increase in gonotrophic cycle (GC) length [46]. Because female tsetse produce unusually few offspring (6–8) over the course of their lifespan (compared to other insects), an increase in GC length would likely result in a significant reduction in population size over time. Infection with a trypanosome strain that induces a metabolically costly immune response also lengthens tsetse’s GC by a duration similar to that (approximately 2 days) induced by infection with *Spiroplasma* [48]. Mathematical modelling indicates that this increase in GC length would theoretically reduce tsetse fecundity by approximately 30% over the course of a female’s reproductive lifespan [48]. Interestingly, most field data indicate a moderate trypanosome infection prevalence of 26%, which is similar to the *Spiroplasma* infection prevalence observed in field captured *Gff* (5–34%, depending on population geographic location) [42, 45]. The models predict that infection prevalence with microbes above these frequencies could significantly decrease fly population size and result in a population crash [48]. In various insects, *Spiroplasma* infection confers pathogen (e.g., nematodes, fungi and parasitoid wasps) resistant phenotypes through the production of immune effector molecules and/or through nutrient scavenging that limits metabolically critical nutrients for other pathogens [49–52]. Our studies with the *Gff* line in which 50% of adults are infected with S*piroplasma* showed a negative correlation between the presence of this bacterium and trypanosome infection success [45]. A similar observation on trypanosome infection success reduction was reported in natural *Gt* populations in West Africa [44]. The mechanism that underlies *Spiroplasma* enhanced refractoriness to trypanosome infection in *Gff* is currently unknown.

The objective of this study was to acquire a better understanding of the mechanism(s) underpinning the enhanced parasite refractoriness noted in *Gff*, including intrinsic fly specific and *Spiroplasma* regulated factors. For our analyses, we used the *Gff* lab line with a heterogenous *Spiroplasma* infection prevalence, and performed high-throughput RNA sequencing of midgut tissue collected from *Spiroplasma* infected (*Spi*^+^), *Spiroplasma* uninfected (*Ctrl*) and only trypanosome (no *Spiroplasma*) infected (*Tbb*^+^) individuals. We describe the host immune and metabolic responses triggered by the presence of either *Spiroplasma* or trypanosomes and incriminate them as factors that limit parasite colonization success. We also discovered a small peptide uniquely present in the genomes of the tsetse species within the *Palpalis* subgroup. This peptide, designated *Gff*Stomoxyn, is related to the Stomoxyn, first described in the insect *Stomoxys calcitrans*. We investigate the spatial and temporal regulation of *GffStomoxyn* expression, its genomic context in tsetse species from different *Glossina* subgroups, and its microbicidal activity against bacteria and BSF and insect stage procyclic (PCF) trypanosomes *in vitro*. We also report on the role of *Gff*Stomoxyn in trypanosome colonization success *in vivo*, using functional studies based on dsRNA mediated gene silencing. We discuss how *Spiroplasma* infection and the Stomoxyn peptide may function to restrict parasite transmission in *Gff*.

## Results

We generated over one billion high quality reads obtained across 14 samples comprising five, five and four biological replicates that represent *Spiroplasma* infected (denoted as *Spi*^+^), *Trypanosome* infected (denoted as *Tbb*^+^), and uninfected (denoted as *Ctrl* with no *Spiroplasma* or trypanosome infection) groups, respectively (S1A Fig). We obtained a Pearson correlation coefficient > 0.7 between the replicates in each experimental condition indicating that the libraries were of high related. Based on the predicted *Gff* transcriptome (*Gff* 2018 reference genome version 63), 84% of known genes were detected with ≥ 10 reads mapping in at least 50% of the biological replicates for each experimental condition (S1A Fig and S2 Table Sheet 1 and 2). These mapping statistics suggested that the majority of the *Gff* transcripts were captured, giving us confidence for a robust downstream analysis.

### Tsetse gut response to infection with symbiotic *Spiroplasma* or parasitic trypanosomes

**Fig 1:**
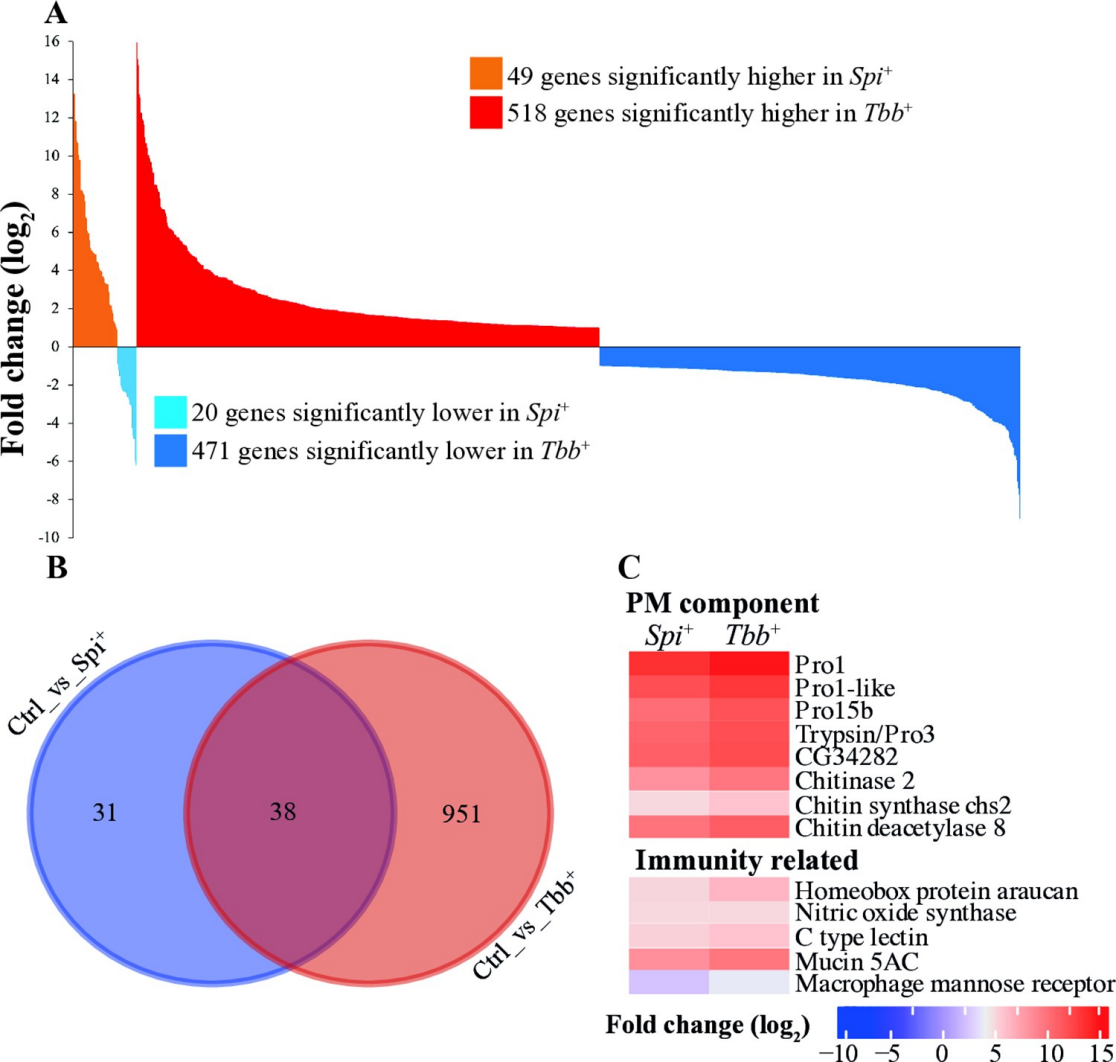
Tsetse Gut Tissue Responses to *Spiroplasma* and Trypanosome Infection. **A**. Differentially expressed (DE) genes identified in the *Spi*^+^ and *Tbb*^+^ groups relative to uninfected *Ctrl* are presented as follows: The orange and red areas represent up-regulated genes in the *Spi*^+^ and *Tbb*^+^ state respectively, while light blue and dark blue indicate down-regulated genes in *Spi*^+^ and *Tbb*+ respectively, at a significance level of 10% adjusted *p*-value and log_2_ fold change (log_2_FC) ≥ 1. **B.** Venn diagram illustrates the number of DE genes that are unique to or shared between the *Spi*^+^ and *Tbb*^+^ states relative to uninfected controls (*Ctrl)*. **C.** Heat maps display the DE genes with putative PM-associated and immunity-related functions that are shared between the *Spi*^+^ and *Tbb*^+^ datasets. Fold-change values are represented as a fraction of the average normalized gene expression levels from age-matched *Spi*^+^ or *Tbb*^+^ versus uninfected control flies (*Ctrl*). The heat maps (dendrograms) were clustered using Euclidean distance calculation methods in R-package software. The clusters were manually separated into various categories.

To assess the impact of *Spiroplasma* or trypanosome infection outcomes on midgut functions, we performed transcriptional comparisons between the *Spi*^*+*^ or *Tbb*^+^ groups relative to the uninfected *Ctrl* group. Infection with *Spiroplasma* resulted in a total of 69 differentially expressed (DE) genes, of which 49 were induced and 20 were suppressed (Fig 1A; S2 Table Sheet 3). In contrast, infection with *Trypanosoma* resulted in a total of 989 DE genes, of which 518 were induced and 471 suppressed (Fig 1A; S2 Table Sheet 4). Clustering based on the Euclidean distances between *Ctrl* and *Tbb*^*+*^ samples resulted in a separate cluster group while no cluster groups were observed between *Ctrl* and *Spi*^*+*^ samples (S1B Fig). These results indicate that infections with trypanosomes induced a more robust response in the gut in comparison to that elicited in the presence of *Spiroplasma* infections.

We next evaluated the DE genes from the *Spi*^*+*^ or *Tbb*^+^ groups to understand the functional impact of each microbe infection on host physiology. We began by investigating the potential functions of the 38 genes (Fig 1B), of which 33 were upregulated and five were downregulated in both *Spi*^*+*^ and *Tbb*^+^ groups compared to control (*Ctrl)* group. Of note, among the induced genes, eight encoded proteins associated with the function of the peritrophic matrix (PM) in tsetse flies. These included four *peritrophins*, *chitin synthase* 2 (*chs*2), a *chitin binding protein* (*CG34282)*, *chitinase* 2, and *chitin deacetylase* 8 (Figs 1C and S2A). Tsetse fly PM is composed of a chitinous matrix embedded with glycoproteins, serving as a barrier that separates the gut lumen from the epithelia. This structure protects the gut cells from harmful compounds present in the bloodmeal as well as from ingested pathogens [53]. The eight induced putative products noted above have previously been described with functions related to PM structure and development in *Gmm* [24] as well as with chitin production or degradation processes [54–56]. Increased expression of genes encoding PM proteins, including Peritrophins (*Pro1* and *Pro3/trypsin)*, has also been observed in trypanosome infected *Gpg* [57]. We previously noted compromised PM function(s) in *Gmm* infected with trypanosomes in the gut and salivary glands as well as in newly eclosed young adults following a bloodmeal supplemented with trypanosomes [58, 59]. Because our results indicated variations in the expression of products involved in PM functions in *Spi*^*+*^ individuals relative to *Ctrl*, we investigated the status of PM integrity by using entomopathogen *S*. *marcescens* in a fly survival assay as we previously reported [24, 58–60]. Typically, flies with compromised PM survive longer as their gut epithelia can detect the presence of *S*. *marcescens* and elicit an immediate and robust response that eliminates the pathogen before it causes a fatal systemic infection [24]. We did not observe a statistically significant difference in host survival between *Spi*^*+*^ and *Ctrl* individuals (S2B Fig), suggesting that the presence of *Spiroplasma* does not influence the structural integrity of PM in the tsetse fly. It is possible that the increased expression of PM associated genes in *Spi*^+^ and *Tbb*^+^ individuals may represent a process of enhanced PM degradation in the presence of pathogens and hence the higher levels of PM associated gene expression to produce more PM proteins to accommodate this process.

**Fig 2:**
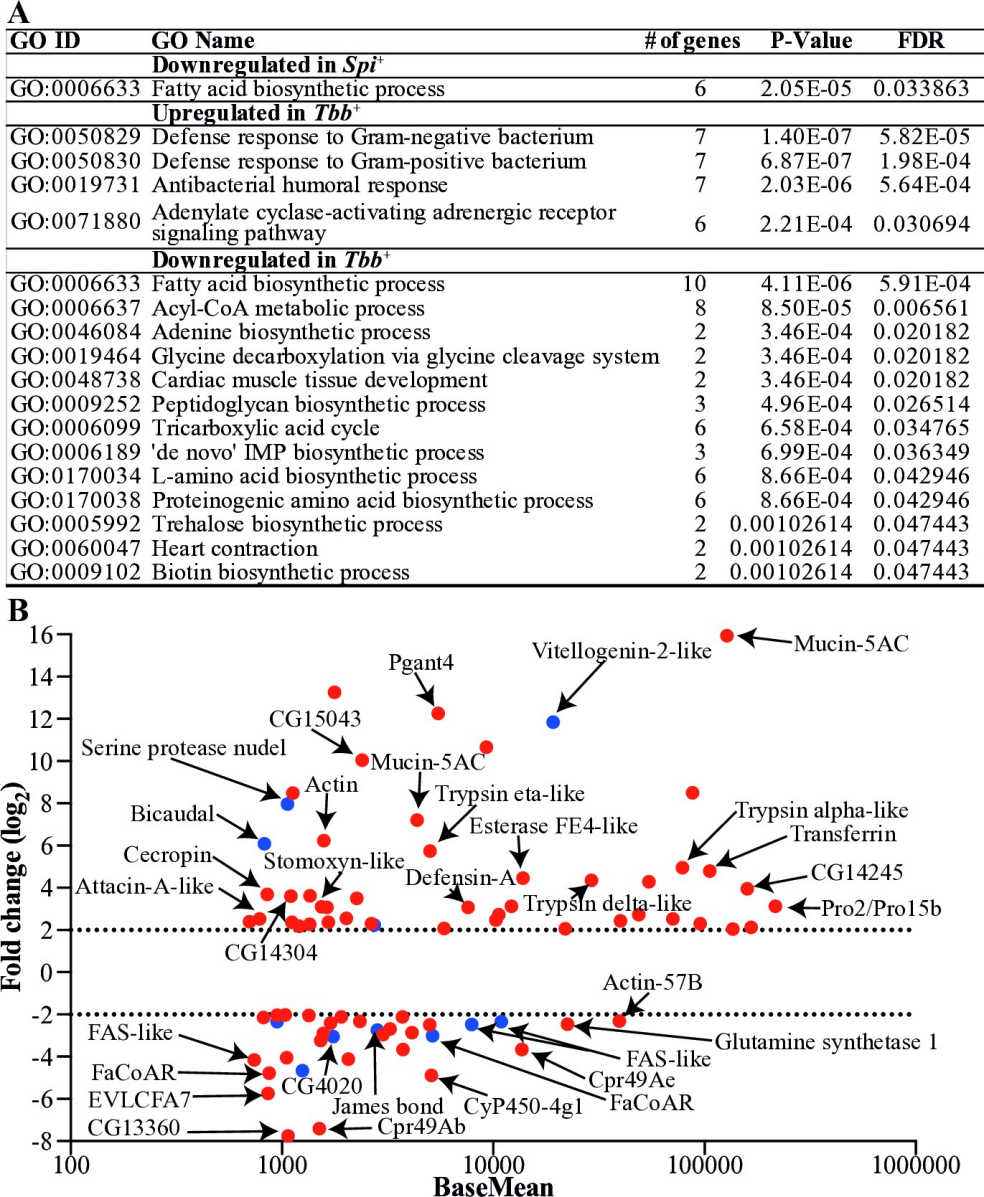
Functional classification of DE-pathways and abundant DE genes unique in *Spi*^+^ or *Tbb*^+^ state. **A.** Gene ontology (GO) enrichment analysis for biological processes was conducted with unique DE genes. **B.** Functional annotations are provided for some of the most abundant and highly DE genes unique to each microbidal infection state, defined by normalized counts ≥ 700 and (log_2_FC) ≥ 2. In this represenetation, red dots indicate genes that are DE in the *Tbb*^*+*^dataset, while blue dots indicate genes that are DE in the *Spi*^*+*^ dataset.

In addition to PM-related functions, we detected immunity-related genes that were induced in both *Spi*^*+*^ and *Tbb*^+^ transcriptome datasets compared to *Ctrl*, including C-type lectin, *mucin-5AC* (GFUI18_012666), and *nitric oxide synthase* (NOS) (Figs 1C and 2SA). These products have been shown to be part of the immune response to trypanosome infections in *Gmm* [41], with the reactive oxygen species, nitric oxide (NO) generated by NOS, exhibiting potent trypanocidal activity [61, 62]. It is possible that the increased expression of these immune molecules in the presence of *Spiroplasma* may contribute to the enhanced parasite refractoriness observed in *Spi*^+^ individuals.

We also detected DE genes unique to each infection: 31 in the *Spi*^*+*^ group and 951 in the *Tbb*^+^ group (S2 Table Sheet 3 and 4). To gain a comprehensive knowledge on the biological process(es) affected by *Spiroplasma* or trypanosome infections, we subjected the microbe-specific DE genes (log_2_FC ≥ 1) to gene ontology (GO) enrichment analysis using Blast2GO software. The GO enrichment analysis of downregulated putative products in both the *Spi*^+^ and *Tbb*^+^ datasets revealed a shared pathway linked to metabolic processes, particularly lipid biosynthesis,. This was supported by the presence of several genes encoding fatty acid synthases (FAS), fatty acyl CoA reductases, and Acyl-CoA binding protein (Figs 2A and S3A). Interestingly, the genes associated with the suppressed fatty acid biosynthesis pathway (GO:0006633) were among the most abundant and highly DE (normalized counts ≥ 700 and log_2_FC ≥ 2) products in both the *Spi*^+^ and *Tbb*^+^ datasets (Figs 2B; S3A). A reduction of lipid availability in the gut could negatively impact host fitness and may also reduce pathogen survival. Despite the decreased expression of lipid-associated genes, we found three transcripts including *carnitine O-palmitoyltransferase* I, *fatty acyl-CoA reductase wat like*, and *james bond* that were upregulated in the *Tbb*^+^ dataset (S3B Fig). Additional biological processes significantly suppressed in the *Tbb*^+^ state included the acyl-CoA metabolic process, the tricarboxylic acid cycle, and amino acid metabolism (Fig 2A).

### Trypanosomes, but not *Spiroplasma* infections, induce canonical immune pathways in *Gff*

Among the genes induced by trypanosomes were those encoding products associated with immunity and adenylate cyclase signaling pathways (Fig 2A). In fact, some of the most abundant and highly DE genes (normalized counts ≥ 700, log_2_FC ≥ 2) in the *Tbb*^+^ group included components of the Immune Deficiency (Imd) pathway, mucins, serine proteases, and redox balance associated proteins (Figs 2B and S3B). In contrast, these immunity related genes were not induced in the *Spi*^+^ group. The induced immunity-related genes in the *Tbb*^+^ group included two peptidoglycan recognition proteins (PGRPs) -*PGRP-*3 and *PGRP-LA-* along with various antimicrobial peptides (AMPs) such as *Attacin A like*, three *Cecropins*, *Defensin* A and Toll (*Toll-like receptor 7*), defense protein l(2) 34Fc, mucins, C-type lectin 37Db and two phenoloxidase 2. We also identified two genes encoding Homeobox family transcription factors, *Wingless* and *Araucan*, associated with the Wingless signaling pathway (S3B Fig). In *Gmm*, this pathway has been implicated in the regulation of expression of host *microRNA-275*, which in turn modulates PM-associated gene expression during the trypanosome colonization process [58]. Further gene families upregulated by trypanosomes included trypsins, six serine proteases (SPs), and two serine protease inhibitors (SPIs) linked with insect immunity (S3C and S3D Fig). SPs and SPIs play critical roles in modulating the expression of immune pathways (Toll and Imd) by regulating the activation of specific effectors following exposure to an infectious agent [63]. Such regulation ensures that the impact of protease-activated cascades remains localized in time and space [64]. Finally, we detected genes associated with detoxification processes in the *Tbb*^+^ group, including nine *cytochrome P450* (*CYPs*) transcripts, six of which were induced and three reduced. In the *Spi*^+^group, we detected three *CYPs* that were DE, with two significantly induced and one reduced relative to controls (S3E Fig). The increased expression of *CYPs* in both the *Spi*^+^ and *Tbb*^*+*^ datasets may suggest a protective response to the heightened oxidative stress caused by the presence of trypanosomes and *Spiroplasma*.

### Impact of *Spiroplasma* and/or trypanosome infection on *Gff* lipid metabolism

**Fig 3:**
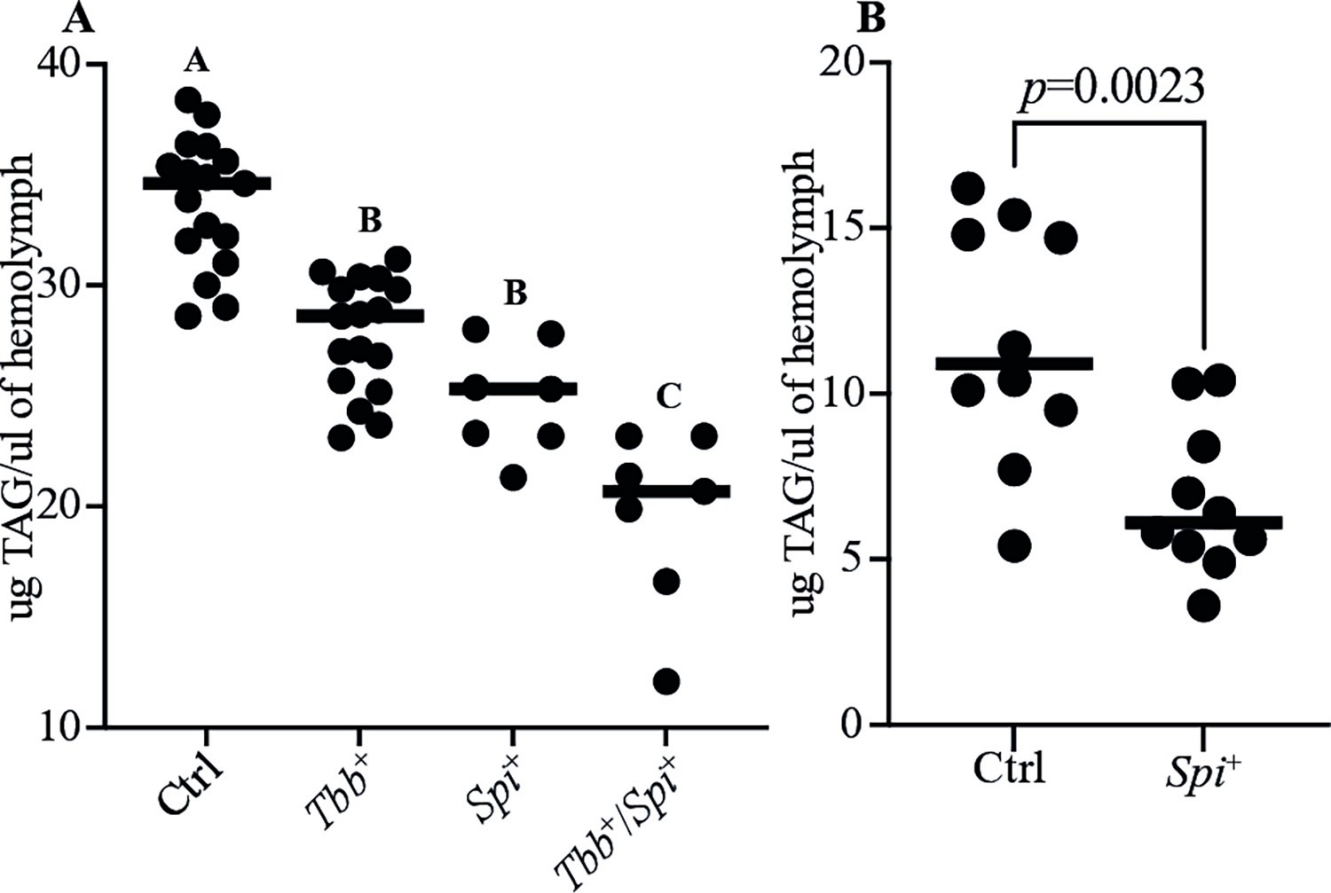
Impact of *Spiroplasma and/or Trypanosome* Infection on Host Lipid Level. **A.** Levels of triacylglyceride (TAG) circulating in the hemolymph of virgin female *Gff* were measure for Control (*Ctrl)*, *Spi*^+^, *Tbb*^+^ and *Spi*^*+*^*Tbb*^*+*^ groups. Letters A, B and C indicate significant differences (*p*<0.05) in TAG levels, while the same letter indicates no significant difference (*p*>0.05). **B**. The amount of TAG in the hemolymph of *Spi*^+^ and Control (*Ctrl)* male *Gff* flies. Statistical significance was determined using ANOVA for virgin female flies and unpaired t-test for male flies.

Our transcriptomic data indicate that infection with *Spiroplasma* and/or trypanosomes negatively impacts the expression of several genes associated with fatty acid biosynthesis, suggesting decreased synthesis and thus low levels of lipids critical for host physiology. We previously demonstrated that pregnant female *Gff* infected with *Spiroplasma* had significantly lower levels of circulating triacyl glyceride (TAG) compared to age-matched, pregnant *Spiroplasma*-negative flies [46]. These flies exhibited reduced fecundity [46], likely as the result of low levels of circulating TAG that make up an important component of tsetse milk [65]. Trypanosomes also scavenge nutrients from their environment, including lipids for metabolism and structural integrity [47, 66]. This nutrient competition may affect critical physiological processes of the host, such as reproductive fitness as previously reported in *Gmm* [48]. With this in mind, we repeated the experiment to investigate the impact of trypanosome infection, and *Spiroplasma* plus trypanosome infections (*Spi*^+^/*Tbb*^+^) on circulating TAG levels in virgin female *Gff*. We used virgin females in this analysis to exclude any effects on TAG levels that might be due to the varying stages of pregnancy. We compared TAG levels in the hemolymph of two-week-old virgin *Spi*^+^, *Tbb*^+^ and *Spi*^+^/*Tbb*^+^ females with their age-matched *Ctrl* females. Our results showed that virgin *Ctrl* females had higher levels of circulating TAG in their hemolymph (33.75±1.005 μg/μl) compared to their age-matched *Tbb*^+^ (27.72±1.438 μg/μl, *p* = 0.0001), *Spi*^+^ (24.90±1.316 μg/μl, *p*<0.0001) and *Spi*^+^/*Tbb*^+^ (19.59±1.438 μg/μl, *p*<0.0001) counterparts (Fig 3A). In addition, we also found that *Spiroplasma*-negative (*Ctrl*) *Gff* males present with significantly more (11.56±1.316 μg/μl) circulating TAG than do males infected with the endosymbiont (6.78±1.316 μg/μl, *p* = 0.0023) (Fig 3B). These findings indicate that the tsetse fly, *Spiroplasma*, and African trypanosomes compete for this metabolically critical TAG. This tripartite interaction likely underlies the reduced fecundity exhibited by *Spi*^+^ females [46], and may account at least in part for why flies that house this bacterium are more refractory to infection with trypanosomes.

### A newly discovered AMP is expressed in the tsetse fly species in the *Palpalis* subgenera

**Fig 4:**
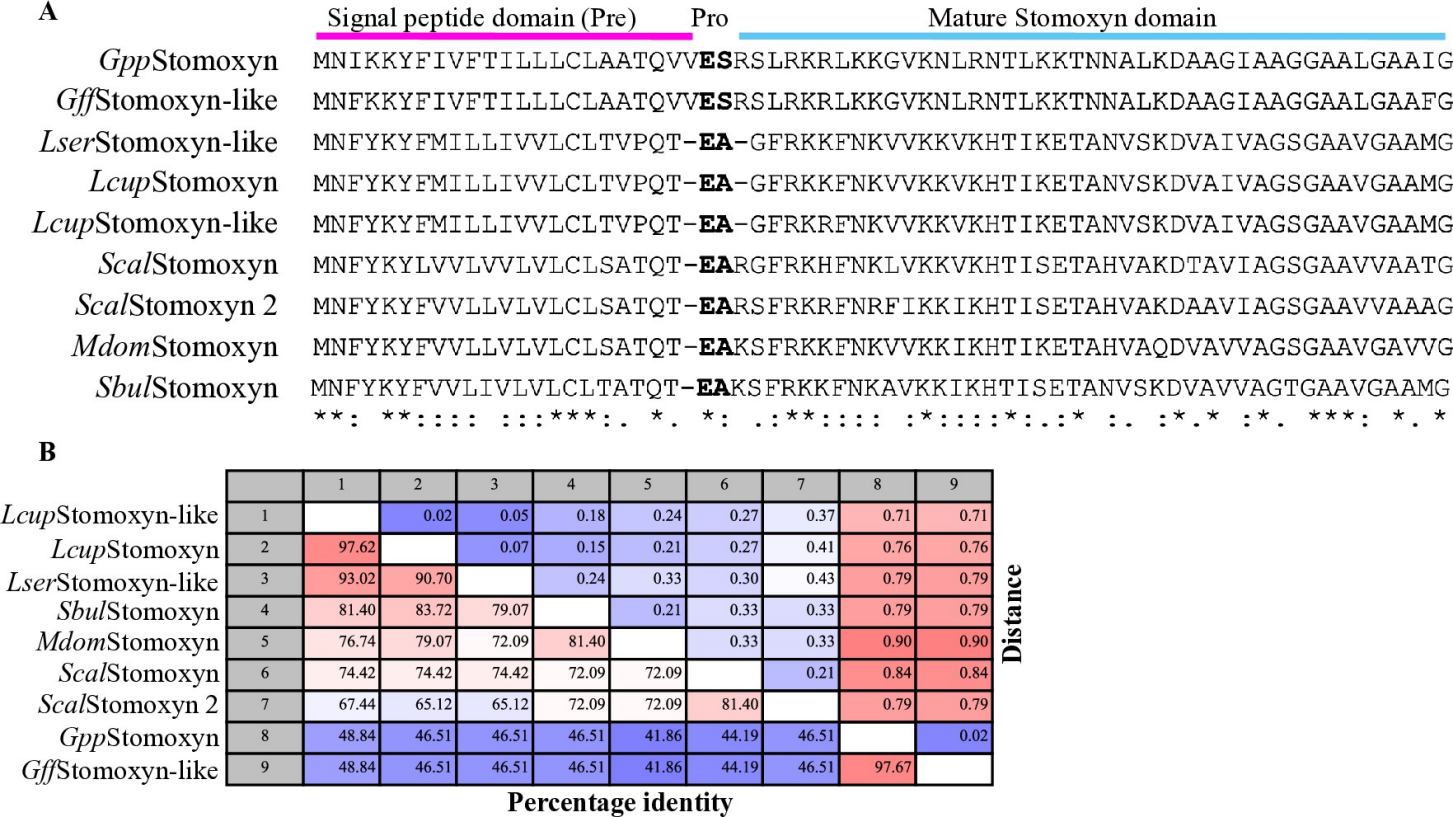
Molecular characterization of *Gff*Stomoxyn. **A.** Sequence alignment of putative Stomoxyn peptides was performed, including sequences from *S*. *calcitrans* (*Scal*Stomoxyn; SCAU016937 and *Scal*Stomoxyn 2; SCAU016907), *Gff (Gff*Stomoxyn-like; GFUI18_001176*)*, *Gpp (Gpp*Stomoxyn*; GPPI027903*), *M*. *domestica* (*Mdom*Stomoxyn-like*; MDOA008330*), *L*. *cuprina* (*Lcup*Stomoxyn; KAI8119624.1 and *Lcup*Stomoxyn-like; XP_023308701.2), *S*. *bullata* (*Sbul*Stomoxyn; DOY81_004902) and *L*. *sericata* (*Lser*Stomoxyn-like; XP_037825072.1). The proteolytic cleavage site is shown in bold letters. The alignment indicates the pre-pro-mature domains of the full predicted peptides, with identical residues indicated by and asterisk (*). Conservative substitutions are marked with a colon (:) and semi-conservative substitutions are marked by a period (.). **B.** Distance matrix analysis was conducted with the mature peptide domains of Stomoxyns from different Diptera.

One of the abundant and DE genes in the *Tbb*^*+*^ dataset was annotated as *Stomoxyn-like* (GFUI18_001176 or GFUI020894-RA), hereinafter referred to as *GffStomoxyn* (Fig 2B). The ortholog of *Gff*Stomoxyn was identified in related Diptera, including *S*. *calcitrans* (stable fly, designated as *Scal*Stomoxyn), *Lucilia sericata* (green blowfly) and *Hermetia illucens* (black soldier fly) [67–69]. The 207 bp *GffStomoxyn* gene encodes a 68 amino acid (aa) pre-pro-mature peptide, which is composed of a 25 aa signal peptide at the N-terminus, followed by pro-mature peptides of 43 aa, similar in organization to the previously described Stomoxyns (Fig 4A). BLASTP homology searches of the 43 aa *Gff*Stomoxyn mature peptide against proteome databases of *S*. *calcitrans* and *Musca domestica* (housefly) identified two orthologs in *S*. *calcitrans*, (SCAU016907; E-value = 8.70E−10 and SCAU016937; E-value = 0.000632 annotated as *Scal*Stomoxyn 2 and *Scal*Stomoxyn, respectively) and a single ortholog in *M*. *domestica* (MDOA008330; E-value = 1.42E-13 annotated as *Stomoxyn-like* but hereinafter referred to as *Mdom*Stomoxyn). We further searched for the *stomoxyn* locus in other tsetse species using the WGS data from *Gpp* in the *Palpalis* subgroup, *Gmm* and *Gpd* in the Morsitans subgroup, *Gau* in the Austenina subgroup, and *Gbr* in the Fusca subgroup. This search revealed a single ortholog in *Gpp* (GPPI027903; E-value = 1.48E−40), while no orthologs were identified in the other four tsetse species. Additionally, we searched the non-redundant (nr) protein sequence database [70] and relevant literature for putative Stomoxyns. This search identified one ortholog in the flesh fly *Sarcophaga bullata* (DOY81_004902), the previously reported one in *Lucilia sericata* (XP_037825072.1; [68]), and two in the Australian sheep blowfly *L*. *cuprina (*XP_023308701.2; KAI8119624.1) (Fig 4A). Interproscan analysis of all identified peptides confirmed their classification within the Stomoxyn protein family. Furthermore, our search also revealed Stomoxyn orthologs in several other Diptera, including *Episyrphus balteatus* (XP_055851874.1) and *Eupeodes corollae* (XP_055904620.1) hoverfly species and the black soldier fly (*H*. *illucens*) (XP_037911389.1; XP_037913598.1) [69].

Multiple sequence alignment of the putative Stomoxyn peptides revealed a conserved structure comprised of pre-pro-mature domains. It is interesting to note that the signal peptide domain of *Glossina* Stomoxyn consists of 25 amino acids, while all other Stomoxyns have a 24-aa signal peptide. This difference includes an additional valine residue and a substitution of alanine with serine at the predicted cleavage site (Fig 4A). Distance matrix analysis of the mature peptide domains showed high identity among the homologs, with *Lcup*Stomoxyn and *Lcup*Stomoxyn-like sharing 97% identify. In contrast, the correspondig domains in the *S*. *domestica* orthologs, *Scal*Stomoxyn and *Scal*Stomoxyn 2, exhibited only 81% identity. Orthologs within closely related species showed high amino acid identity, such as *Gff* and *Gpp* at 97.67% and *L*. *sericata* and *L*. *cuprina* at 93.02% (Fig 4B). The amino acid identity within the mature peptide domain of Stomoxyns between *M*. *domestica* and *S*. *calcitrans* was 72%, while *Gff*Stomoxyn and *Gpp*Stomoxyn exhibited 46% identity with the orthologs from *M*. *domestica* and *S*. *calcitrans* (Fig 4B). Phylogenetic analysis of mature stomoxyns’ amino acid sequences indicate that the *Glossina* stomoxyn-like loci cluster together and are sister to the stomoxys clade, derived from other muscoid flies (S4A Fig). This stomoxyn phylogeny coincides with host nuclear phylogenies [71], suggesting that further sampling across *Calyptrate* taxa would yield additional stomoxyn-like peptides. I-TASSER prediction of the putative tertiary structure for mature *Gff*Stomoxyn and *Scal*Stomoxyn 2 indicate that they are composed of double α-helices and β-folds **(**S4B Fig), similar to the structure reported for *Scal*Stomoxyn [72].

We analyzed the genomic context flanking the *Stomoxyn* loci in *Gff* and *Gpp* and compared their synteny using WGS data available for other *Glossina* species, where no orthologs were identified (S4C Fig). This analysis revealed several syntenic loci located immediately upstream and downstream of the *Stomoxyn* locus in both *Gff* and *Gpp*. Notably, several loci *GffStomoxyn* were retained on the same genomic scaffolds in *Gpd*, *Gau* and *Gbr*, while such syntenic loci were largely absent from *Gmm* WGS data. Additionally, a BLASTP search of the pre-pro-mature *Gff*Stomoxyn sequence against the putative proteomes of different tsetse species did not yield any significant hits, further suggesting that this protein-coding sequence is absent in those species. To rule out the possibility that the absence of the *Stomoxyn* locus was due to inadequate genome annotation, we performed *de novo* transcript assembly from midgut RNA-seq datasets available for *Gmm* and *Gpd* [58, 73] using Trinity software [74]. We then performed BLASTN searches with the *stomoxyn* sequences collated from *Gff*, *Gpp*, and other Diptera against our transcriptome-based assembly. This search also did not yield significant hits, further confirming the absence of this locus in *Gmm*, *Gpd*, *Gau* and *Gbr*.

We next investigated the presence of the *stomoxyn* locus in several *Gff* individuals obtained from distinct populations in North-West Uganda, as well as in *Gpg* from West Africa, *Gbr* and *Gau* from South Africa and *Gau* and *Gpd* from Kenya. We also analyzed this locus in a colony of *Gpp* that was distinct from the one used for the WGS analysis. PCR-based amplification of the *stomoxyn* loci—which included 5’ and 3’ UTRs, two exons and one intron–followed by sequence analysis of the products revealed over 99% identity at the nucleotide level between field collections and flies from laboratory lines. This high level of identity was also observed among *Gff*, *Gpp* and *Gpg* indicating the close evolutionary relatedness of these fly species (S4D Fig).

### Analysis of *stomoxyn* expression profile and regulation

**Fig 5:**
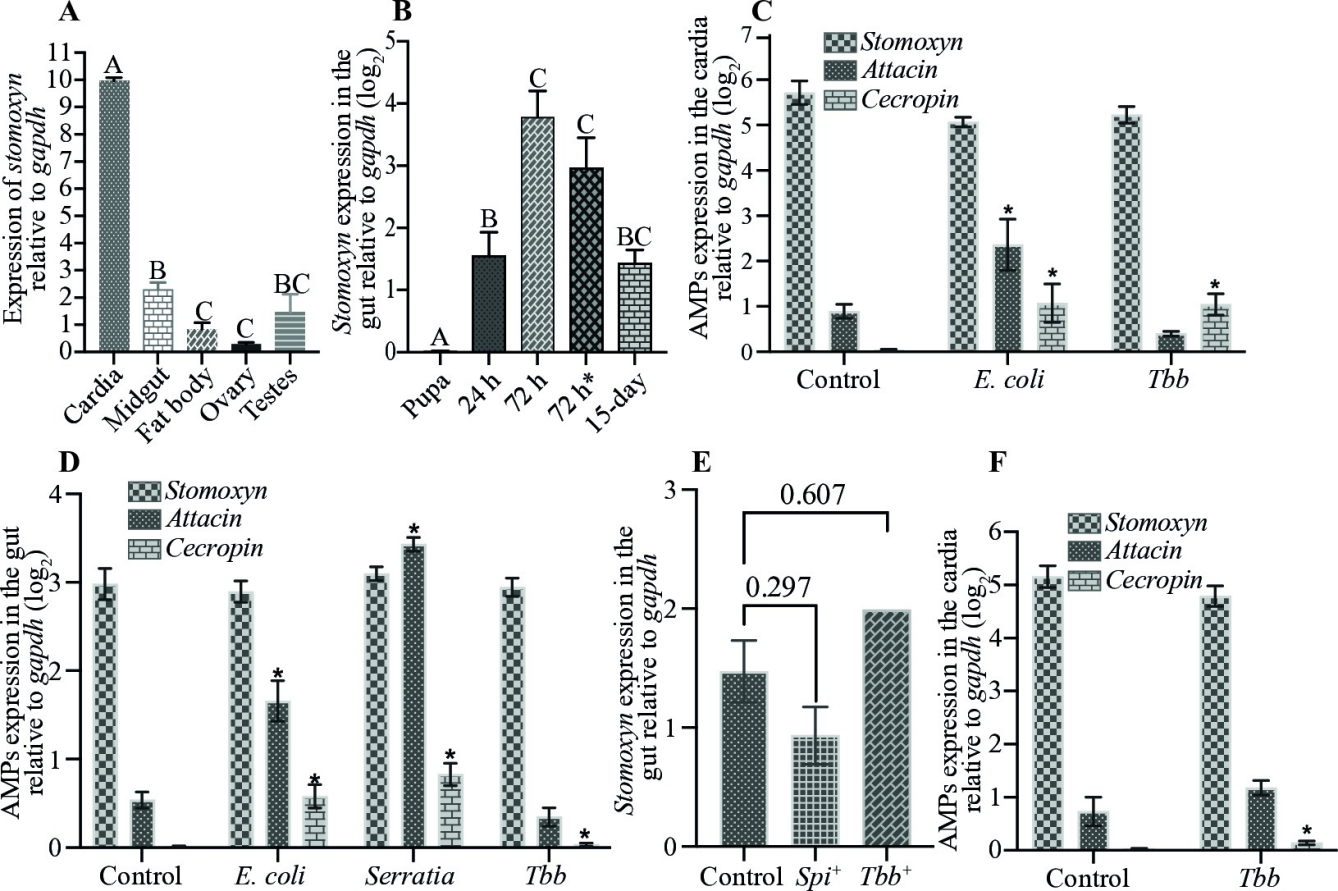
Spatial and Immune Responsive Expression of *GffStomoxyn*. **A.** The qRT-PCR expression profile of *stomoxyn* was analyzed from the cardia, midgut, fat body, female ovary, and male testes tissues. Eight biological replicates were used, each consisting of three individual fly tissues collected from 10-day old adults. Results are presented relative to *gapdh* expression. Letters A, B and C above each bar represent significant differences in *stomoxyn* expression (*p*<0.05), while the same letter indicates no significant difference (*p*>0.05). **B.** Temporal expression of *stomoxyn* was measured from late pupa and whole gut tissue at multiple time points: 24 and 72 h post-eclosion, 72 h after the first bloodmeal (designated as 72hr*), and 15 day-old adult flies analyzed 72 h after last bloodmeal. Six to ten biological replicates, each comprised of individual midguts, were used in the experiment. Results are presented relative to *gapdh* expression. Letters A, B and C on top of each bar indicate significant differences (*p*<0.05) in *stomoxyn* expression, while the same letter indicates no significant difference (*p*>0.05).

We profiled *stomoxyn* expression across various tissues, including cardia, midgut, fat body, female ovary and male testes. Our results indicate that *stomoxyn* is preferentially expressed in the cardia, with significantly lower expression detected in the midgut and other tissues (Fig 5A). *Stomoxyn* has been similarly reported to be preferentially expressed in the anterior midgut region of *S*. *calcitrans* [67]. Next, we examined the temporal expression profile of *stomoxyn* and found that gut transcript levels increased in adults post-eclosion, peaking at 72 h, when newly eclosed flies are mature enough to imbibe their first bloodmeal. Gut *Stomoxyn* levels measured 72 h after the first bloodmeal and following multiple bloodmeals remained equally high (Fig 5B). We also evaluated the expression of three antimicrobial peptides (AMPs), *stomoxyn*, *cecropin* and *attacin*, in the cardia of adult flies 72 h after their last bloodmeal. Similarly, we evaluated the expression of these AMPs in the cardia following systemic stimulation by *E*. *coli* or *per os* stimulation by a BSF *Tbb*-containing bloodmeal. Our results revealed significantly higher levels of *stomoxyn* expression in the cardia compared to the other AMPs and its expression remained high but unresponsive to immune stimuli (Fig 5C). The levels of both *cecropin* and *attacin* showed a significant increase following *E*. *coli* challenge, while only *cecropin* was significantly increased following trypanosome challenge (Fig 5C). We also evaluated the inducible nature of AMP expression in the gut (midgut and cardia) following immune stimulation of teneral *Gff* adults by *per os* challenge with *E*. *coli*, *S*. *marcescens*, and BSF *Tbb*. While expression of *cecropin* and *attacin* was significantly induced by *E*. *coli* and *S*. *marcescens*, *stomoxyn* expression remained high but unchanged in the gut (Fig 5D), similar to our findings in the cardia tissue. To see if *Spiroplasma* infection prevalence influenced *stomoxyn* levels, we quantified the *stomoxyn* expression in the gut of 15-day old adults infected with either *Spiroplasma* (*Spi*^*+*^) or *Trypanosomes* (*Tbb*^*+*^) and compared it to that found in uninfected control (*Ctrl)* guts. Results indicated that *stomoxyn* expression measured in the gut was not influenced by either *Spiroplasma* or trypanosome infection status of the host (Fig 5E). Additionally, using *Gpg*, another member of the *Palpalis* subgroup of tsetse flies, we measured the expression level of *stomoxyn* in the cardia of adult flies and compared its transcript levels to *attacin* and *cecropin* following immune challenge with a *Tbb*-supplemented bloodmeal (Fig 5F). We found that *stomoxyn* transcript levels were comparable between *Gff* and *Gpg* cardia tissues, and were significantly higher than those of the other two AMPs in the unchallenged state (Control). Similarly, we found that neither *stomoxyn* nor *attacin* were induced upon trypanosome challenge in the cardia in *Gpg*, while *cecropin* expression increased upon trypanosome challenge similar to our findings in *Gff*. Collectively, our results indicate that *stomoxyn* is expressed at high levels predominantly in the cardia tissue of both teneral and mature adult tsetse. This finding is consistent with previous reports of *stomoxyn* expression in *S*. *calcitrans*, where it was shown to localize to the anterior gut [67]. Unlike canonical AMPs, such as *attacin* and *cecropin*, which are induced in response to pathogen exposure, *stomoxyn* is constitutively expressed in the cardia, and is not responsive to microbial presence, similar to the expression pattern reported in *S*. *calcitrans* [67].

**C.** The expression levels of *stomoxyn*, *attacin* and *cecropin* were analyzed in the cardia tissue of 8-day old adult *Gff* flies, 72 h after their last bloodmeal. The last bloodmeal for the *E*. *coli* and *Tbb* groups was supplemented with *E*. *coli* and *Trypanosome (Tbb)*, respectively, while the Control group received a normal bloodmeal. This experiment included five biological replicates, each comprising three cardia tissues dissected from individual flies. *Tbb* indicates trypanosome challenge. *indicates significantly different gene expression (*p*<0.05) compared to the unchallenged control. **D.** The expression levels of *stomoxyn*, *attacin* and *cecropin* in the gut of newly eclosed flies were analyzed 24 h following a bloodmeal supplemented with *E*. *coli*, *Tbb* or *Serratia*, and compared to controls that received a normal bloodmeal. This experiment included nine to twelve biological replicates, each consisting of individual flies. *Tbb* indicate trypanosome challenge. *indicates significantly different gene expression (*p*<0.05) relative to the unchallenged control. **E.** The relative expression of *stomoxyn* was measured in the guts of 15-day old *Spi*^+^ or *Tbb*^+^ flies, and compared to age-matched uninfected controls. This experiment included eight to twelve biological replicates, each comprised of individual fly guts dissected 72 h post-bloodmeal. *Trypanosome* or *Spiroplasma* infection status of each fly was determined as described in the Materials and Methods section. *Tbb*^+^ indicates trypanosome infected *Gff* (*Spiroplasma* uninfected); *Spi*^+^ indicates *Spiroplasma* infected (Trypanosome uninfected). P-values for each comparison are shown above the corresponding bar. **F.** The relative expression levels of *stomoxyn*, *attacin and cecropin* were determined in the cardia tissue of 8-day old *Gpg* flies, 72 h after their third bloodmeal supplemented with trypanosomes. This experiment included five biological replicates, each comprising three cardia from individual flies. *Tbb* indicate trypanosome challenge. *indicates significantly different gene expression (*p*<0.05) relative to the control.

### Antimicrobial activity spectrum of *Gff*Stomoxyn

**Fig 6:**
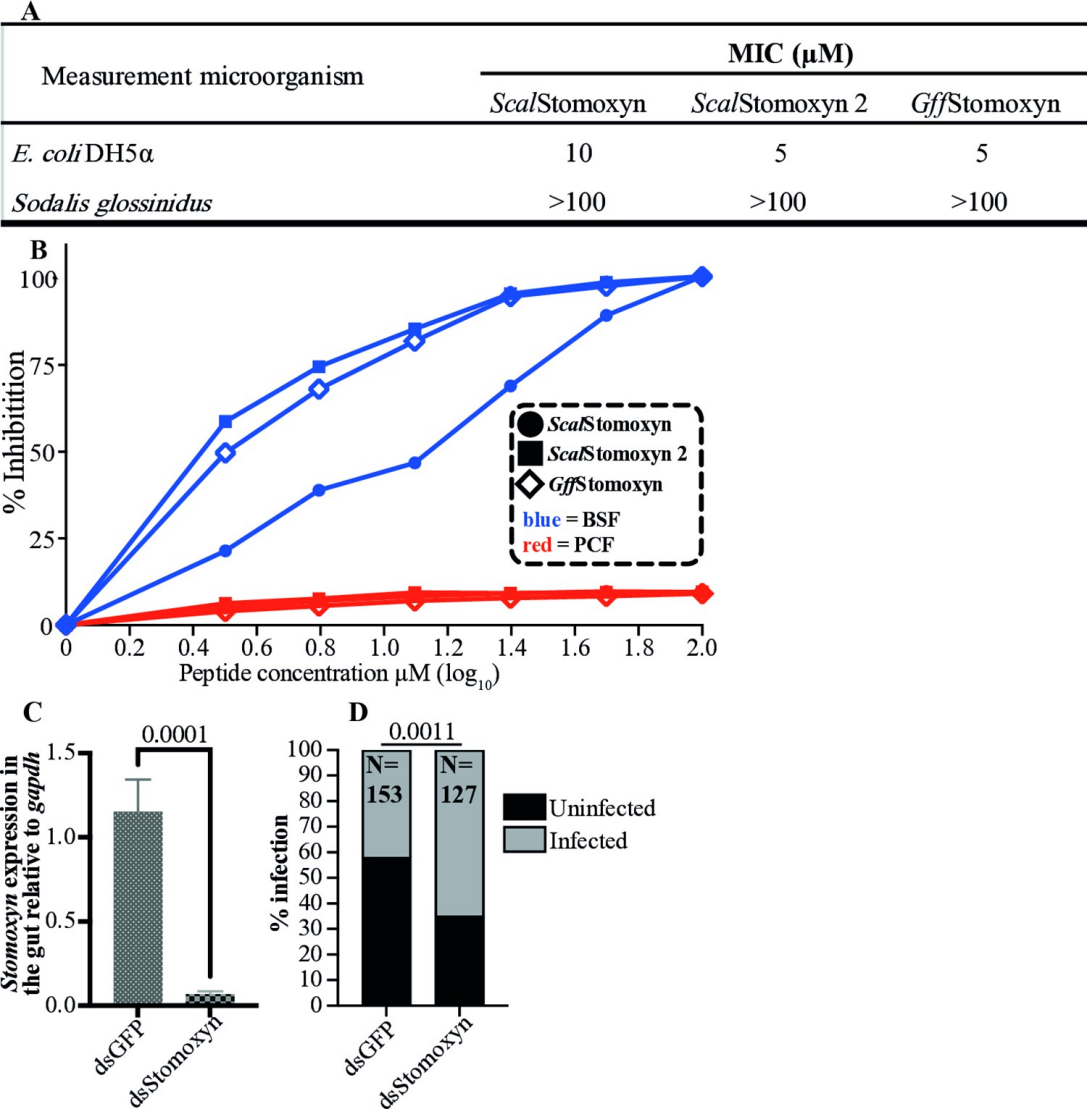
*In vitro* and *in vivo* Bioactivity of Stomoxyn. **A**. *In vitro* antibacterial activity: The antibacterial activity of mature *Scal*Stomoxyn, *Scal*Stomoxyn 2 and *Gff*Stomoxyn peptides was assessed against *E*. *coli* and *Sodalis* bacteria. **B.** *In vitro* bioactivity against trypanosomes: The trypanocidal activity of mature *Scal*Stomoxyn, *Scal*Stomoxyn 2 and *Gff*Stomoxyn peptides was assessed against mammalian bloodstream forms (BSF) and insect stage procyclic form (PCF) trypanosomes. The blue lines indicate BSF parasite inhibition and the red lines show PCF parasite inhibion over the range of peptide concentrations tested. Different recPeptides are shown by varying symbols. **C.** Gene-silencing validation: The relative expression of *stomoxyn* was measured from dsGFP and dsStomoxyn*-*treated *Gff* flies, showing a significant decrease in expression (*p*<0.0001) in the case of dsStomoxyn*-*treated group. Ten biological replicates, each comprising individual flies, were used to validate silencing efficacy. **D.** Prevalence of trypanosome infections: The prevalence of midgut trypanosome infections in dsRNA-treated *Gff* flies was microscopically analyzed 15 days post-parasite acquisition, with significant difference observed (*p*<0.0011). The total number of flies (N) used in each experimental condition is shown. The experiment included a mixture of male and female flies across two biological replicates, with no significant difference in infection rates between the dsRNA-treated males and females.

We next investigated whether *Gff*Stomoxyn exhibits trypanocidal activity similar to that reported for *Scal*Stomoxyn [67]. We commercially generated synthetic mature *Gff*Stomoxyn, and mature *Scal*Stomoxyn [67] as well as its ortholog, *Scal*Stomoxyn 2. We assessed the microbicidal activity of these three synthetic peptides against *E*. *coli*, *Sodalis* (the tsetse fly commensal symbiont) and *Tbb* (both BSF and PCF forms) *in vitro* using minimum inhibition concentration (MIC) assays. The MIC values for *Scal*Stomoxyn, *Scal*Stomoxyn 2 and *Gff*Stomoxyn against *E*. *coli* were 10 μM, 5 μM and 5 μM, respectively. None of the three peptides exhibited activity against *Sodalis* at concentrations up to the 100 μM (Fig 6A), consistent with the previously published results for *Scal*Stomoxyn [67, 75]. In contrast, the minimum median inhibitory concentration (MIC_50_) values for *Scal*Stomoxyn, *Scal*Stomoxyn 2 and *Gff*Stomoxyn against BSF trypanosomes were 18.4, 2.01 and 2.51 μM, respectively (Fig 6B and replicated in S5 Fig). These results indicate that *Scal*Stomoxyn 2 exhibits significantly stronger killing activity against BSF trypanosomes compared to previously identified *Scal*Stomoxyn (at 2.01 and 18.4 μM, respectively). *Gff*Stomoxyn also demonstrated a high level of trypanolytic activity, with an MIC_50_ of 2.51 μM (Fig 6B and replicated in S5 Fig). However, all three peptides were ineffective against PCF trypanosomes with the peptides being able to kill less than 10% of the parasites at concentrations up to 100 μM (Fig 6B and replicated in S5 Fig). Our results indicate that *Gff*Stomoxyn exhibits strong antimicrobial activity against both gram-negative *E*. *coli* and BSF trypanosomes *in vitro*. Conversely, this peptide is not effective against the endosymbiotic *Sodalis* and insect stage PCF trypanosomes, both of which have coadapted to survive in the insect midgut environment.

### *Stomoxyn* decreases susceptibility of tsetse fly to trypanosome infections

We employed an RNAi-based silencing approach to investigate the influence of Stomoxyn peptide on parasite establishment in *Gff*. Double-stranded RNAs, including dsGFP and dsStomoxyn, were injected into the thoracic haemocoel of teneral adult flies. Fourty-eight hours after treatment, we confirmed that *GffStomoxyn* expression in dsStomoxyn-treated flies was significantly reduced compared to the dsGFP treated group (*p*<0.0001; Fig 6C). At this time point, both dsGFP and dsStomoxyn flies were offered bloodmeal containing 1x10^6^ BSF trypanosomes and subsequently maintained on a normal blood diet. This experiment was replicated twice. On day 15, we analyzed gut parasite infection prevalence from two replicates and combined the for subsequent analysis. Chi-square analysis revealed that infection rates in the dsStomoxyn group were significantly higher (82/127; 64.56%) compared to the dsGFP -treated flies (64/153; 41.83%) at df = 1, X^2^ = 10.63, *p* = 0.0011 statistical level of significance (Fig 6D). These findings indicate that Stomoxyn exhibits trypanocidal activity against BSF trypanosomes *in vivo* in the gut, similar to the effects observed with synthetic peptides *in vitro*.

## Discussion

Understanding the determinants of vector competence in different tsetse species and subgroups can be complex. These factors may include genetic differences, as revealed by available WGS data, as well as extrinsic factors, such as resident microbiota and the varying ecological niches they occupy. Here, we focused on one of the most important vector species from the genus *Glossina*, *Gff*, which has a wide distribution in sub-Saharan Africa. Together with closely related species in the *Palpalis* subgroup, such as *Gpp* and *Gpg*, *Gff* is responsible for over 90% of Human African Trypanosomiasis (HAT) cases. Although *Gff* are prolific vectors of HAT pathogens as they inhabit areas close to human settlement, water bodies and are highly anthropophilic, laboratory studies indicate they also exhibit strong resistance to gut parasite colonization and parasite transmission [13, 76]. Our findings identify an important genetic factor present in *Gff* and other *Palpalis* subgroup species but absent in tsetse species from other subgenera: the immune peptide Stomoxyn. This peptide exhibits strong antiparasitic activity *in vitro* against the mammalian BSF trypanosomes that get acquired through infectious bloodmeal. Functional studies in which we reduced the expression of *Gff*Stomoxyn *in vivo* using dsRNA based RNAi treatments showed a higher prevalence of parasite infections compared to control groups treated with dsGFP, supporting our *in vitro* findings with the synthetic *Gff*Stomoxyn peptide. In addition to this genetic factor, our global gene expression profiling of *Spiroplasma-*infected *Gff* individuals suggests that this symbiotic association negatively affects the host’s metabolic capacity, specifically in lipid biosynthesis. Quantification of triacylglycerides in the hemolymph of *Spiroplasma-*infected *Gff* confirmed a reduction, and these lipids may play a crucial role in both trypanosome survival and host fecundity. In conclusion, both the metabolic effects mediated by *Spiroplasma*, which limit critical nutrients, and the high levels of trypanocidal Stomoxyn peptide constitutively expressed in the anterior gut of young adults, contribute to the enhanced resistance to parasites observed in these species.

Using a *Gff* line where *Spiroplasma* infections are maintained in approximately 50–60% of adult individuals, we previously demonstrated a negative correlation between the presence of *Spiroplasma* and trypanosome infection success [45]. Similar results were observed in another species within the subgroup *Palpalis*, *Gt*, collected from Ghana and Burkina Faso [44]. Our comparative transcriptome analyses revealed that infections with *Spiroplasma* symbionts and trypanosome parasites trigger dramatically different host responses in the gut. While we detected nearly 1000 differentially expressed (DE) genes in response to trypanosome infection, only about 70 genes were DE in the presence of *Spiroplasma*. This suggests that trypanosomes are recognized as pathogens, triggering a strong immune response, while *Spiroplasma* endosymbiont behaves more like a commensal organism, eliciting only a minimal host response. Interestingly, this subdued host response to the presence of *Spiroplasma* in the gut is contrary to the strong transcriptional responses we observed in the gonads of tsetse flies where the symbiont influences host reproductive physiology, including sperm viability [46]. However, the stronger host response in the gonads may be due to the higher density of the endosymbiont present in these tissues relative to the gut [42].

A common host response to infection with either trypanosomes or *Spiroplasma* is a reduction in the expression of genes associated with fatty acid synthesis and reduced fatty acid biosynthetic processes, suggesting reduced availability of lipids for physiological functions in hosts infected with these microbes. In the triatomine bug *Rhodnius prolixus*, infection with *Trypanosoma cruzi* also decreases the expression of several genes associated with lipid biosynthesis [77]. Our TAG measurement assay, comparing *Spi*^+^, *Tbb*^+^ and *Spi*^+^/*Tbb*^+^ to the control group uninfected with either microbe (*Ctrl)*, indicated significantly reduced circulating TAG levels in the hemolymph of both virgin female and male *Gff* flies infected with these microbes. These findings mirror our previously reported results where we observed lower levels of circulating triglycerides in the hemolymph of *Spi*^+^ pregnant female *Gff* [46]. It is possible that a reduction in TAG levels could result from *Spiroplasma* and/or trypanosomes scavenging these metabolites as nutrient sources [47, 78], or from decreased transcriptional activity in lipid biosynthesis pathways. Given that lipids are critical for immunity, reproduction and as a source of energy for both the host and its microbial partners [79, 80], decreased lipid levels can negatively impact tsetse fecundity as well as trypanosome physiology and parasite infection success. Collectively our results suggest that both *Spiroplasma* and trypanosome parasites may consume critical nutrients from their host, such as triacylglycerides, thus disrupting metabolic processes essential for host functions, including immunity and reproduction [78, 81].

In the *Tbb*^+^ dataset, the GO analysis of the putatively induced products indicated enrichment of immunity pathways, including *C-type lectins*, *phenoloxidases*, *mucins*, *PGRP*, *tsetseEP* protein and several AMPs. A similar response to *Tbb* infection was reported in the species *Gmm*, where the expression of many key immune-associated genes was induced in various body compartments [22, 25, 59, 62, 67, 82–85]. The functional roles of some of these immune effectors in trypanosome transmission have been validated through RNAi studies, including IMD, *attacin*, *cecropin* [26, 86], *tsetseEP protein* [31] and *PGRP-LB* [35]. Tsetse species that exhibit greater resistance to trypanosome infections, such as *Gpp* and *Gpd*, express higher levels of Phenoloxidase compared to the susceptible *Gmm* [87]. Studies in *Gmm* indicated that parasite exposure induces *nos* resulting in increased trypanolytic nitric oxide (NO) activity as well as an reactive oxygen intermediate (ROI) and hydrogen peroxide (H_2_O_2_) levels in the cardia organ [61, 62]. These outcomes may mediate molecular communications between local and systemic responses to clear parasite infection. In contrast, *Spiroplasma* infection did not significantly modulate the expression of genes associated with known canonical immune pathways, except for an increase in *nos* levels, which may confer additional parasite resistance to *Spiroplasma*-infected *Spi*^+^ flies. A previous study in *Drosophila melanogaster* also found that *Spiroplasma* infection did not induce epithelial immune responses [88]. This subdued response could result from the absence of a cell wall structure in *Spiroplasma* which typically contains immune-activating Pathogen-Associated Molecular Patterns (PAMPs) needed to trigger antimicrobial responses [89, 90].

Besides the metabolic effects conferred by *Spiroplasma*, we also report the discovery of a highly expressed gene in *Gff* encoding trypanocidal Stomoxyn, the orthologue of *Scal*Stomoxyn, which was first identified in the stable fly, *S*. *calcitrans*. Stomoxyns are structurally similar to the Cecropin family of antimicrobial peptides, which are linear and amphipathic in nature with an α-helical structure that lacks cysteine residues. *Gff*Stomoxyn is produced as a pre-pro-mature peptide and has the predicted 3D α-helix structure similar to that of *Scal*Stomoxyn [72]. We discovered that *Glossina* Stomoxyns contain an additional valine residue in the signal peptide, and a serine substitution at the signal peptide cleavage site. It remains to be determined whether these amino acid differences impact the protein’s structure or its interaction with the signal peptidase enzyme, which is responsible for cleavaging the propeptide to its mature form. Future biochemical studies are required to shed light on the structural variations observed at the genomic level.

Our genomic investigations indicate the presence of a single stomoxyn locus in *Gff*, *Gpp*, *M*. *domestica* and *L*. *sericata*, while the genomes of *S*. *calcitrans*, *L*. *cuprina*, and *H*. *illucens* each contain two copies of this locus, with the homologs showing over 90% identity [68, 69, 91–93]. These homologs may represent recent gene duplication events given that they are more closely related in sequence to one another than they are to their orthologs across taxa, and they appear to be located on the chromosome as tandem copies. Despite multiple lines of investigations, we found that the *stomoxyn* locus is absent in flies from different subgenera of *Glossina*, including laboratory lines of *Gmm*, *Gpd* and *Gbr* as well as natural populations of *Gpd*, *Gau* and *Gbr*. Prior investigations using cDNA and gDNA from *Gmm* also failed to detect the presence of a *stomoxyn-like* gene in this species [67]. However, the homolog of *GffStomoxyn* is present in the genome of *Gpp* in a region sympatric with *Gff*, suggesting a common ancestor. Although WGS for other species within the *Palpalis* subgroup are yet to be generated, PCR-amplification and sequence analysis indicate that the *stomoxyn* ortholog is also present in *Gpg*. Investigations of *Gff* and *Gpg* obtained from Uganda and Mali have confirmed its presence in natural populations. Hence it appears that *stomoxyn* is a subgroup-specific gene within the genus *Glossina*. Given that the *Palpalis* subgroup represents a recent expansion in the evolution of *Glossina*, it remains to be determined whether the ancestral lineages lost the s*tomoxyn* locus or if the species within the *Palpalis* subgroup acquired it post-speciation. Initial analysis of the contigs containing the *stomoxyn* locus for potential flanking mobile element-like sequences did not reveal any candidates that could suggest a potential mechanism of acquisition. Further studies are necessary to investigate how this gene locus was acquired exclusively by this *Glossina* subgroup.

In *S*. *calcitrans*, *stomoxyn* is constitutively expressed in the anterior gut, and due to its trypanocidal activity, may confer trypanosome resistance in this hematophogus insect, which is phylogenetically related to *Glossina* [67]. We also found *GffStomoxyn* to be preferentially and constitutively expressed in the cardia organ of *Gff* in the anterior gut. Since the expression of insect AMPs, such as *attacin* and *cecropin*, is known to be induced upon detection of microbial PAMPs by the immune system shortly after infection, we investigated whether *GffStomoxyn* expression might also be immune-responsive. In our transcriptome data, we observed an increase in *GffStomoxyn* expression in the *Spi*^+^ and *Tbb*^+^ datasets, although the induction by *Spiroplasma* was below the level of statistical significance (*p* = 0.1268, adjusted p = 0.584 for *Spi*^+^; *p* = 0.0318, adjusted p = 0.089 for *Tbb*^+^). Similarly, our qPCR experiments did not show a significant induction of *GffStomoxyn* expression in the gut following *per os* challenge with *E*. *coli* or trypanosomes, nor in *Spiroplasma*-infected *Gff*. This lack of validation may be partly due to the high variability observed in expression levels across the different biological replicates. Such variation could stem from the spatial and temporal nature of *GffStomoxyn* expression, which is preferentially localized to the small cardia organ in the anterior gut and exhibits an increasing expression profile over the 72 h post-bloodmeal acquisition.

Although the mature *Gff*Stomoxyn peptide exhibits only 45–50% identity with other Stomoxyns, it has retained its microbicidal and trypanocidal activity. In our *in vitro* assays with synthetic peptides, *Gff*Stomoxyn had a stronger microbicidal activity than the previously described *Scal*Stomoxyn and was similar to that of the *Scal*Stomoxyn 2 ortholog discovered in the *S*. *calcitrans* genome. However, varying purity levels of the synthetic peptides may have contributed to this discrepancy and as such requires further investigations. Functional studies with Stomoxyns from *S*. *calcitrans*, *H*. *illucens* and *L*. *sericata* also indicated broad-spectrum activity against Gram-negative and Gram-positive bacteria, as well as fungi [67–69, 75, 93]. Our studies with synthetic *Gff*Stomoxyn and *Scal*Stomoxyn2 indicated that both peptides are effective against the mammalian BSF trypanosomes with similar LD_50_ values, but are not effective against the insect stage procylic (PCF) parasites. In addition, *Gff*Stomoxyn showed high antibacterial activity against *E*. *coli* but not against the *Sodalis* endosymbiont, mirroring earlier reports of *Scal*Stomoxyn activity [75]. Experiments assessing the density levels of the symbionts *Sodalis* and *Wigglesworthia* in *Spiroplasma*-infected *Gff* also showed no reductions compared to control *Gff* [46]. Functional experiments in which we successfully reduced *GffStomoxyn* in the cardia of teneral flies via RNAi found a significant increase in gut trypanosome infection prevalence in the ds*Gff*Stomoxyn group relative to control dsGFP groups, confirming that *Gff*Stomoxyn is active *in vivo*. We hypothesize that *Gff*Stomoxyn may play a crucial role and can be one of the factors that interfere with parasite viability shortly after acquisition in an infected-bloodmeal. This interference likely occurs before the BSF parasites transform into PCF cells within a few days, at which point they are rendered resistant to the trypanolytic actions of this peptide, even at high concentrations.

In conclusion, our results indicate that the reduced vector competence associated with *Gff*, which is enhanced when the fly houses *Spiroplasma*, could arise from both unique genomic content of the species within this subgroup of tsetse and competitive nutritional dynamics between the vector and its symbiont. The reduced lipid production in *Spiroplasma*-infected *Gff* likely limits the availability of essential nutrients required to maintain metabolic hemostasis for both the vector and trypanosome parasites. The exact molecular pathways through which *Spiroplasma* suppresses host lipid biosynthesis remain unclear. A better understanding of how these mechanisms persist or evolve over time could provide a more complete picture of parasite-host dynamics. It remains to be seen whether *Spiroplasma* infections impair reproductive fitness of its host in natural populations, potentially contributing to imperfect vertical transmission from mother to offspring and resulting in the sporadic infection prevalences reported in the field. In addition to metabolic interference, host immune responses to *Spiroplasma*, such as the increased expression of NOS, may elevate oxidative stress levels in the anterior gut, resulting in lethal effects on BSF parasites acquired through the infected bloodmeal. The high levels of trypanolytic Stomoxyn peptide, constitutively produced in the cardia organ located in the anterior gut, may eliminate BSF parasites early in the infection process, before BSF parasites differentiate into Stomoxyn-resistant PCF cells that colonize the gut. Future experiments aimed at the mechanistic basis of Stomoxyn-mediated BSF parasite clearance during early infection process, as well as the potential role of *Spiroplasma* in regulating Stomoxyn expression in the cardia, could expand our understanding of the tripartitite host-symbiont-parasite dynamics.

Given that *Sodalis*, the tsetse fly commensal endosymbiont, is resistant to the microbicidal actions of Stomoxyn, it may be feasible to develop genetically modified *Sodalis* lines that express Stomoxyn. The ability to colonize tsetse flies with modified *Sodalis*, an approach called “paratransgenesis”, could facilitate the development of parasite-resistant flies. Introducing these modified flies into field populations could effectively replace their naturally susceptible counterparts thereby reducing disease transmission [94]. However, applying these strategies to wild tsetse populations may present several challenges. These include the presence of sympatric tsetse species in many disease endemic regions, the necessity for vertical transmission of the paratransgenic organism, and the development of systems that enable modified symbionts to spread among flies in natural populations. One potential early application of this approach could be in the ongoing Sterile Insect Technique (SIT) programs aimed at tsetse fly elimination [95]. Because the male tsetse flies used in SIT releases are also potential disease vectors, colonizing them with modified *Sodalis* expressing Stomoxyn could bolster parasite resistance in sterile-males and enhance the efficacy of the releases and success of vector control applications.

## Material and methods

### Biological material

*Glossina fuscipes fuscipes* (*Gff*) pupae were obtained from the Joint FAO/IAEA IPCL insectary in Seibersdorf, Austria and reared at Yale University insectary at 26°C with 70–80% relative humidity and a 12 hour light:dark photo phase. This *Gff* line carries the heterogenous symbiotic infection, with about 50% of flies being negative (uninfected) for *Spiroplasma*. Newly eclosed teneral *Gff* females were challenged with 2x10^6^ *Trypanosoma brucei brucei* RUMP 503 strain BSF parasites (referred here as *Tbb*) per ml of blood in their first bloodmeal and were hereafter maintained on defibrinated bovine blood every 48 h through an artificial membrane feeding system [96] for 15 days. Another group of the *Gff* flies received normal bloodmeals, were maintained under the same conditions and used for uninfected controls. Whole gut (comprising cardia and midgut) were dissected 48 h after the last bloodmeal in Phosphate-buffered-Saline-Glucose (PSG) buffer (pH 8.0) and trypanosome infection status was microscopically determined using a Zeiss Axiostar Plus light microscope (Carl Zeiss Microscopy GmbH, Jana, Germany). All dissected tissues were stored individually at -80°C for RNA extractions. Legs corresponding to individual flies were also collected and kept separately at -80°C for genomic DNA (gDNA) production and determination of *Spiroplasma* infection status.

### Genomic DNA extraction and *Spiroplasma* infection determination

The legs obtained above from individual flies were used to extract gDNA using the Monarch Genomic DNA purification kit following manufacturer’s protocol (New England BioLabs, MA, USA). A *Gff* specific *alpha*-*tubulin* primer set was used for gDNA quality control, and *Spiroplasma*-specific 16s rDNA primer set was used to determine the infection status of the bacterium in each fly. The *Spiroplasma* locus was amplified using touchdown PCR as previously described [45]. Based on the PCR results for *Spiroplasma* infection status, the samples were pooled for downstream analysis as either *Spiroplasma* uninfected (*Ctrl*: control negative for *Spiroplasm*a) or *Spiroplasma* infected (*Spi*^*+*^; positive only for *Spiroplasma*). Flies that were provided a bloodmeal supplemented with *Tbb* and microscopically found to be infected as described above were similarly screened for the presence of *Spiroplasma*. Of the trypanosome positive flies, only those that were *Spiroplasma* negative were used for downstream analyses (*Tbb*^+^, positive only for trypanosomes). Primer sequences and PCR-amplification conditions used as in S1 Table.

### RNA extraction, RNA-seq library preparation and Bioinformatics analysis

Total RNA was extracted from the gut of all adult individuals using TRIzol and subsequently treated with Turbo-DNase to eliminate contaminating DNA following the protocol described by the manufacturer (Thermo Fisher Scientific Inc., CA, USA). Elimination of DNA from the RNA was confirmed by PCR amplification using *Gff-*specific *alpha*-tubulin and glyceraldehyde-3-phosphate dehydrogenase (*gapdh*) primer sets (S1 Table). RNA quantity and quality was determined using an Agilent 2100 Bioanalyzer RNA Nano chip (Agilent, Palo Alto, CA, USA). RNA from two individuals was pooled per biological replicate and four, five and five biological replicates representing *Ctrl*, *Spi*^*+*^, and *Tbb*^+^ groups, respectively were obtained.

RNA-seq libraries were prepared using the NEBNext Ultra RNA Library Prep Kit for Illumina (New England BioLabs, Inc., MA, USA) according to the manufacturer’s protocol. The individual libraries were barcoded for Illumina HiSeq 2500 sequencing system (Illumina, Inc., CA, USA) and paired-end sequenced (100 bases) at Yale Center for Genome Analysis (YCGA, New Haven, CT). Read files are deposited in the National Center for Biotechnology Information (NCBI) archive, BioProject ID PRJNA1112339.

Raw RNA-seq reads were checked for quality using FASTQC and parsed through Trimmomatic for quality trimming [97]. For analysis, the *Gff* 2018 reference genome version 63 was obtained from VectorBase [98] (https://www.vectorbase.org/). The quality filtered reads were aligned using STAR (v2.3) [99] and ‘htseq-count’ function in the HTSeq (v0.11.2) [100] was used to count the number of reads mapped per gene with the intersection-nonempty mode. Using counts data from HTSeq, correlation between replicates within each condition was evaluated by calculating Pearson’s correlation coefficient (*r)* value. Only genes that had ≥ 10 reads mapping in at least 50% of the biological replicates for each experimental condition were used for downstream analysis. DESeq2 [101] was then used to determine differentially expressed (DE) genes between the control and the infected groups. Genes were considered significantly DE if they exhibited log_2_ fold change either ≥ 1 or ≤ -1 at a *p* value < 0.05 and an adjusted *p value* < 10% [84, 102]. Gene ontology (GO) enrichment analysis of the DE genes was performed using the Blast2GO software [103] using the blastx [104] algorithm at significance threshold of 1×10^−3^ to search against non-redundant (NR) NCBI protein database. An enrichment analysis via Fisher’s exact test at an FDR, p-value ≤ 0.05 in Blast2GO was conducted to determine overexpressed GO terms, relative to the entire *Gff* transcriptome.

### Investigation for Peritrophic Matrix integrity

To assess the effect of *Spiroplasma* infection on the PM integrity, we made use of a host survival assay following infection of the *Gff* line described above with *Serratia marcescens* strain db11 [24, 58]. Briefly, newly eclosed teneral flies were provided a bloodmeal supplemented with *S*. *marcescens* (1×10^3^ CFU/mL). All flies were then maintained on normal bloodmeals and the number of fly deaths were recorded every 48h. Dead flies at each collection date were kept individually at -80°C for subsequent assessment of *Spiroplasma* infection status via the PCR amplification assay described above. This assay was repeated twice.

### Hemolymph Triacylglycerol (TAG) assay in *Spiroplasma* of Trypanosome infected *Gff*

Hemolymph (3 μl/fly) was collected as described in [105] from two-week-old *Ctrl*, *Spi*^+^, *Tbb*^+^ and *Spi*^+^/*Tbb*^+^ virgin female *Gff*, centrifuged (4°C, 3000xg for 5 minutes) to remove bacterial cells, diluted 1:10 in PBS containing 1.2 μl/ml of 0.2% phenylthiourea (to prevent hemolymph coagulation) and immediately flash frozen in liquid nitrogen [46]. These experiments used two-week-old flies, as this is the typical timepoint for assessing trypanosome infection status by microscopy. Unmated virgin flies were chosen for this analysis because triacylglycerol (TAG) is produced in tsetse’s fat body and exported into the hemolymph, where it is taken up by the milk gland and incorporated into milk secretions. Since TAG is the most abundant lipid in tsetse milk, even small differences in pregnancy stage (i.e., several hours) can significantly affect circulating TAG levels. By using virgin flies, were were able to eliminate pregnancy-related variations and focus on the effects of *Spiroplasma* or trypanosome infection. Hemolymph TAG levels were quantified colorimetrically by heating samples to 70°C for 5 min followed by a 10 min centrifugation 16,000xg. Five μl of the supernatant was added to 100 μl of Infinity Triglycerides Reagent (Thermo Scientific) and samples were incubated at 37°C for 10 min. Absorbance was measured at 540nm using a BioTek Synergy HT plate reader as described [46, 106]. Male *Gff* flies *Ctrl* and *Spi*^+^ were also included in the experiment and treated the same way. All *Gff* sample spectra data were compared to that generated from a triolein standard curve (0–50 μg, 10 μg increments). Analysis of Variance (ANOVA) was employed in the analysis of the amount of TAG circulating in the hemolymph of *Ctrl*, *Spi*^+^, *Tbb*^+^ and *Spi*^+^*/Tbb*^+^ female and t-test for circulating TAG in *Gff* male flies’ hemolymph using GraphPad Prism software v.7 (GraphPad Software, La Jolla CA, USA).

### Genomic context and structural analysis of Stomoxyn peptides

To confirm the VectorBaseDB [98] annotation of *Gffstomoxyn-like* gene (GFUI18_001176 or GFUI020894-RA), Blastp [104] homology searches were performed using the putative proteomes for *Stomoxys calcitrans* and *Musca domestica* [92, 107]. This analysis revealed one ortholog in *Glossina palpalis palpalis* (*Gpp*Stomoxyn, GPPI027903), the previously reported Stomoxyn peptide in *S*. *calcitrans* annotated as *ScalStomoxyn* (SCAU016907) [67] as well as an additional ortholog (annotated as *ScalStomoxyn 2*, SCAU016907) and a single ortholog in *M*. *domestica* (annotated as *Stomoxyn-like* MDOA008330). Search of published literature and analysis of NR database identified the presence of other orthologs in related Diptera, including *Sarcophaga bullata* (DOY81_004902), *Lucilia sericata* (XP_037825072.1), two in *Lucilia cuprina (*XP_023308701.2 and KAI8119624.1), *Episyrphus balteatus* (XP_055851874.1), *Eupeodes corollae* (XP_055904620.1) and two in *Hermetia illucens* (XP_037911389.1; XP_037913598.1). Full length protein sequence alignment of all significant Blast hits using clustalW [108] and InterProScan analysis [109] were performed to determine conserved Stomoxyn structural and functional domains. RAxML-NG with LG+G8+F model, with 25 parsimony, 25 random starting trees and 1000 bootstraps [110] was used to develop phylogenic tree based on MAFFT [111] sequence alignement of the mature Stomoxyn domain. The resulting tree was visualized with FigTree (http://tree.bio.ed.ac.uk/software/figtree/) and comparatively analyzed with that present in the *Gff* and *Gpp* proteome. The tertiary structure of the Stomoxyn peptides were predicted using the web based I-TASSER program Full length protein sequence alignment of all significant Blast hits using clustalW [108] and InterProScan analysis [109].

In order to understand the evolutionary history of this locus across *Glossina*, we obtained the WGS data available for the different tsetse species in VectorBaseDB, and compared the regions flanking the *Gff* and *Gpp Stomoxyn* loci with the same regions from *Gmm*, *Glossina pallidipes (Gpd)*, *Glossina austeni* (*Gau*) and *Glossina brevipalpis* (*Gbr*), which lack *GffStomoxyn* ortholog. To do this, genes sequences flanking the *Gff*Stomoxyn (GFUI18_001176) in *Gff* supercontig JACGUE010000004 were compared with those in *Gpp* KQ080227 supercontig that contains *GppStomoxyn* via Blastp searches [104]. Similarly, Blastp was used to query the putative proteomes of other *Glossina* species for the presence of *Gff*Stomoxyn orthologs and the supercontigs where the ortholog exist in the genome.

The presence of the *stomoxyn* locus was also investigated from natural fly populations, Genomic DNA was prepared from *Gff* individuals collected in 2018 from the Albert Nile river drainage in Northwest Uganda (Amuru district: Gorodona (GOR; 3°15’57.6"N, 32°12’28.8"E), Okidi (OKS; 3°15’36.0"N, 32°13’26.4"E), and Toloyang (TOL; 3°15’25.2"N, 32°13’08.4"E)), *Glossina palpalis gambienses* (*Gpg*) collected from Bado Souleymane Mali (15°10’0”N, 7°31’0”W), *Gbr* and *Gau* from Hells Gate South Africa, *Gau* and *Gpd* from Shimba Hills Kenya. Genomic DNA was also prepared from a colony of *Gpp* maintained in Burkina Faso that is distinct from the colony in Seirbersdorf Vienna used for the WGS analysis and *Gff* colony from Seirbersdorf Vienna. The gDNA PCR primers (S1 Table) were designed to amplify the entire genomic sequence/region of *stomoxyn* in these species. When PCR products were obtained, they were analyzed by Sanger sequence and aligned using CLC Main Workbench (CLC bio, Cambridge, MA) against *Gff* and *Gpp stomoxyn-like* genomic loci from VectorBase.

### Synthesis of Stomoxyn peptides

The predicted mature sequences of three Stomoxyn peptides were commercially synthesized and procured from ABClonal Science (500 West Cummings Park, Woburn, MA). These included the two homologs of Stomoxyn peptide from *S*. *calcitrans*, the previously described Stomoxyn (*Scal*Stomoxyn) [67] and Stomoxyn 2 (*Scal*Stomoxyn 2) discovered here from the *S*. *calcitrans* WGS data, and *Gff*Stomoxyn described here. The synthesized peptide sequences were RSLRKRLKKGVKNLRNTLKKTNNALKDAAGIAAGGAALGAAFG (*Gff*Stomoxyn), RSFRKRFNRFIKKIKHTISETAHVAKDAAVIAGSGAAVVAAAG (*Scal*Stomoxyn 2) and RGFRKHFNKLVKKVKHTISETAHVAKDTAVIAGSGAAVVAATG (*Scal*Stomoxyn). Purity of these synthetic peptides were 85.181%, 89.177% and 90.635%, for *Scal*Stomoxyn, *Scal*Stomoxyn 2 and *Gff*Stomoxyn respectively.

### *In vitro* antibacterial and anti-trypanosomal activity of Stomoxyn peptides

The antimicrobial activity of the synthetic Stomoxyn peptides was tested against *E*. *coli*, tsetse endosymbiont *Sodalis*, and BSF and PCF trypanosomes. For antimicrobial activity, an overnight *E*. *coli* culture was inoculated into fresh medium and grown to logarithmic phase (OD_600_ 0.3–0.4 at 37°C to yield about 1.5x10^8^ cells/ml). A 1:10 dilution of this culture was used to test the killing activity of different Stomoxyn peptide concentrations. Stock solutions (1 M) of synthetic peptides were prepared in water and 2-fold serial dilutions were made corresponding to 10 μM down to 1.25 μM. The *E*. *coli* culture was inoculated with synthetic peptides (0, 1.25, 2.5, 5 and 10μM concentrations) for testing the minimal inhibitory concentration (MIC). Cultures were grown for 1 h at 37°C and plated in duplicate on LB plates which were incubated overnight to measure colony forming units. For *Sodalis* killing assays, the bacteria were cultured on brain-heart infusion agar supplemented with 10% bovine blood as previously described [112] and grown in liquid cultures to OD_600_ 0.3–0.4 at 25°C_._ The synthetic peptides (0μM, 10μM, 20μM, and 100μM concentrations) were prepared and applied as described above for *E*. *coli*. The experiment was repeated three times with each synthetic peptide and microorganism.

The anti-trypanosomal activity of Stomoxyn was determined using Alamar Blue assay as described [67, 113] with slight modifications. Briefly, 500 BSF *Antat 1*.*1* 90:13 *T*. *b*. *brucei* cells in 50 μl were added to 96-well microtiter plates containing 50 μl of culture medium with a 2-fold serially diluted *Stomoxyn* peptide [114]. Top and low peptide concentration were evaluated at 100 and 3.125 μM respectively. The test for each concentration was performed in triplicates. As controls, culture media was included for baseline fluorescence as well as parasites without the Stomoxyn peptide. After 72 h of incubation at 37°C, 10 μl of Alamar Blue was added to each well, and the plates were incubated for another four hours. The plates were then read using BioTek cytation1 imaging reader (Agilent Technologies Inc., Santa Clara, CA, USA) at an excitation wavelength of 530 nm and an emission wavelength of 590 nm [67]. The acquired data was analyzed to produce sigmoidal inhibition curves and to determine IC_50_ (median drug concentration inhibiting 50% of fluorescence development) values. The Stomoxyn percentage inhibition was calculated using the formula: % inhibition = 100[1-(X-MIN)/(MAX-MIN)] where: X was fluorescence of the sample, MIN fluorescence of the control (media without parasites) and MAX fluorescence of the positive control (culture parasites without drug) [115]. Stomoxyn activity for the PCF *Tbb* was determined following the same protocol above using cells grown in SDM-79 medium at 28°C [116].

### Analysis of *GffStomoxyn* expression

Analysis of spatial expression of *GffStomoxyn* was performed on the cardia, midgut, fat body and ovary tissues from females, and testes tissue from males dissected from 10-day old *Gff*. For temporal expression analysis, whole guts, including cardia and midgut, were dissected from three week-old pupae, teneral adults 24 and 72 h post eclosion, adults 72 h after their first bloodmeal and 15-day old adults that have taken multiple bloodmeals were included in the experiment.

We exposed 8-day old *Gff* adults that have had two normal bloodmeals to microbial challenge. The first two groups (Control and *E*. *coli*) were provided a normal bloodmeal, while the third (*Tbb*) received a bloodmeal supplemented with 1×10^6^ BSF *Tbb* per ml of blood. Eight hours after the last bloodmeal, the flies in the *E*. *coli* group were exposed to 1000 CFU *E*. *coli* by intrathoracic microinjection. The cardia of all flies were dissected 72 h after the last bloodmeal, and five biological replicates, each containing three cardia, were obtained for downstream RNA extractions.

To determine the immune responsive profile of *GffStomoxyn* expression, three experimental groups of teneral flies (48 h post eclosure) were provided with bloodmeals supplemented with 1,000 colony forming units (CFU) of *E*. *coli*, or *S*. *marcescens* strain db11, or 1×10^6^ BSF *Tbb* per ml of blood, respectively. A fourth control group was similarly established that received a normal bloodmeal. All flies that did not take the bloodmeals were removed from the experiment and the guts of remaining flies from all four experimental groups were dissected 24 h after exposure for RNA extraction.

For analysis of trypanosome infected adults, *Gff* were provided with 1×10^6^ BSF *Tbb* per ml of blood in their first bloodmeal and subsequently maintained on normal uninfected bloodmeal for 15 days. Forty-eight hours post last bloodmeal, the midgut of these flies dissected and the presence of trypanosome parasites in the gut microscopically determined using a Zeiss Axiostar Plus light microscope (Carl Zeiss Microscopy GmbH, Jana, Germany). Trypanosome infected guts were individually stored at 80°C until RNA extraction. Another group of uninfected control *Gff* flies were similarly treated but maintained on uninfected bloodmeals. Legs corresponding to individual flies were collected and frozen separately at -80°C for gDNA extraction and determination of *Spiroplasma* infection status as described above. Only flies that were determined to be *Spiroplasma* negative (*Ctrl*), or *Spiroplasma* infected (*Spi*^+^) or trypanosome infected but *Spiroplasma* negative (*Tbb*^+^) were used for downstream RNA extractions, subsequent cDNA synthesis and gene expression analysis.

We provided 8-day old *Gpg* adults that have had two normal bloodmeals a third bloodmeal supplemented with 1×10^6^ BSF *Tbb* per ml blood and established a control age-matched group that received a normal bloodmeal. The cardia of all flies were dissected 72 h after the last bloodmeal, and five biological replicates, each containing three cardia, were obtained for downstream RNA extractions.

Total RNA was prepared (and DNase treated) from these samples as described above. cDNA was synthesized with oligo-dT primers and random hexamers using the iScript cDNA synthesis reaction kit (Bio-Rad, Catalog No. 170–8891) according to the manufacturer’s protocol. Real time quantitative PCR (qRT-PCR) *GffStomoxyn* and other genes (S1 Table) were performed in technical duplicate for each sample. The expression level of *GffStomoxyn* was evaluated between tissues and/or experimental conditions by qRT-PCR analysis using *Gff gapdh* as internal control. All qRT-PCR results were thus normalized to tsetse *gapdh*, quantified from each biological replicate in the tissues or experimental condition. The qRT-PCR data was analyzed using Relative Expression Software Tool (REST)-384 version 2 software [117].

### RNAi gene silencing and trypanosome infection prevalence

*Green fluorescent protein* (*gfp*) and *GffStomoxyn* specific dsRNAs were prepared using the MEGAscript High Yield T7 transcription kit (Ambion, Huntingdon, UK) and gene specific dsRNA primers (S1 Table). The PCR products dsGFP and ds*Gff*Stomoxyn were sequenced to confirm their specificity for the gene of interest. To test the efficacy of gene silencing, groups of teneral male and female flies 48 h post eclosion were intrathoracically microinjected with 5 μg ds*Gff*Stomoxyn or dsGFP in 2 μl nuclease-free water and the midguts were dissected 48 h post treatment. RNA was extracted from the dissected guts and analyzed by qRT-PCR amplification for *GffStomoxyn* expression from each treatment group as described above. For trypanosome infection effects, two groups of teneral flies treated with ds*Gff*Stomoxyn or dsGFP as above were provided 1×10^6^/ml BSF *Tbb* in their first bloodmeal administered 48 h post dsRNA treatments. Flies that did not feed were discarded, and all remaining flies were subsequently maintained on normal diets. Fifteen days post-trypanosome challenge, all surviving flies were dissected, and their midguts microscopically examined for the presence of parasite infections. Chi-square was used to compare proportions of trypanosome infections between dsGFP and ds*Gff*Stomoxyn treatment groups. Gene silencing and trypanosome infection experiment was done twice.

## Supporting information

S1 Fig Overview of the Gff Transcriptome. **A.** Table summarizes results obtained from different biological replicates across three conditions, *Ctrl*, *Spi*^+^ and *Tbb*^*+*^. Condition: Ctrl: *Spiroplasma* and trypanosome negative midgut; *Spi*^+^: *Spiroplasma* positive midgut; *Tbb*^+^: trypanosome positive midgut; BRep: Biological Replicate; UMR: Number of Uniquely Mapped Reads to the *Gff* genome (*Gff*_genome-2018_ver 63); No. Trans: Number of expressed transcripts, defined as those with normalized read coverage ≥ 10 in at least 50% of the biological replicates per condition. **B.** Heat map showing the Euclidean distances between biological replicates, calculated from the regularized log transformation of the data, provides insights into the similarities and differences among the conditions.
(TIF)

S2 Fig Differentially Expressed Shared Transcripts and Peritrophic Matrix Integrity. The Heat map denotes the fold changes of differentially expressed (DE) genes that are shared between the *Spi*^*+*^ and *Tbb*^*+*^ states, according to their putative functions. Fold-change values are expressed as a fraction of the average normalized gene expression levels from age-matched *Spi*^+^ or *Tbb*^+^ relative to the control *Ctrl*. The heat maps (dendrograms) were generated using Euclidean distance calculation combined with ward.D clustering methods within the R-package software. The clusters were manually separated into two categories: PM and Immunity Functions. **B.** Effect of *Spiroplasma* infection on Peritrophic Matrix integrity. The survival of flies was monitored every 48 h following a *per os* treatment of teneral adult flies with *Serratia marcescens*, administered 72 h post-eclosion. At time of death, the *Spiroplasma* infection status of each fly was evaluated using our diagnostic assay. The Kaplan-Meyer survival curves illustrate the fly survival over time for *Spiroplasma*-uninfected flies (blue) and *Spiroplasma*-infected flies (red). This experiment was conducted twice, with no significant differences observed between the two experiments.
(TIF)

S3 Fig Heatmap representation of unique differentially expressed (DE) transcripts. The heatmaps depict the fold changes of unique DE transcripts across various functional categories, comparing infected transcriptomes and the uninfected control. *Spi*^+^: *Spiroplasma* infected, *Tbb*^+^: trypanosome infected; FC: fold change indicate the degress of change in expression levels relative to uninfected controls; 1: differentially expressed transcripts; NDE: transcripts that are not differentially expressed.
(TIF)

S4 Fig Genomic Characterization of Stomoxyn locus. **A.** Phylogenetic tree of mature Stomoxyn sequences from nine different Diptera species based the Maximum likelihood (ML) model. Sequences used in this analysis were obtained from VectorBase for *S*. *calcitrans* (*Scal*Stomoxyn; SCAU016937 and *Scal*Stomoxyn 2; SCAU016907), *Gff (Gff*Stomoxyn-like; GFUI18_001176*)*, *Gpp (Gpp*Stomoxyn*; GPPI027903*) and *M*. *domestica* (*Mdom*Stomoxyn-like*; MDOA008330*), and from NCBI database for *L*. *cuprina* (*Lcup*Stomoxyn; KAI8119624.1 and *Lcup*Stomoxyn-like; XP_023308701.2), *S*. *bullata* (*Sbul*Stomoxyn; DOY81_004902), *L*. *sericata* (*Lser*stomoxyn-like; XP_037825072.1), *Episyrphus balteatus* (*Ebal*Stomoxyn-like; XP_055851874.1) and *Eupeodes corollae* (*Ecor*Stomoxyn-like; XP_055904620.1). The analysis involved 11 amino acid sequences and 1000 bootstrap replications. **B.** The tertiary structure of the mature *Gff*Stomoxyn and *Scal*Stomoxyn 2 peptide was predicted by I-TASSER. **C.** Genomic content surrounding the Stomoxyn-like gene (GFUI18_001176) focusing on the supercontig JACGUE010000004 of the Gff genome assembly (version 63, Vectorbase). The supercontigs available from the other *Glossina* WGS data were also compared. Genes exhibiting synteny among different tsetse species are indicated by arrows with the same color genes that do not shown synteny are presented in gray. The black arrow marks the expected location of the *Stomoxyn* gene in *G*. *pallidipes*, *G*. *austeni* and *G*. *brevipalpis*. Gene size and spacings are not drawn to scale. Based on the current assembly of the region, only one *Stomoxyn* gene is present in *Gff* and *Gpp*, while it is absent in other *Glossina* species. **D.** Multiple sequence alignment of the Stomoxyn locus from *Gff*, *Gpp* and *Gpg*. The alignment includes genomic PCR product sequences of the *Stomoxyn* locus from flies obtained from laboratory and field populations, confirming the presence and conservation of this locus in the species from *Palpalis* subgroup. The primers for the PCR amplification were designed to span the entire coding region of the Stomoxyn pre-pro-mature peptide.
(TIF)

S5 Fig Bioactivity of Stomoxyn Against Trypanosomes. A replicate experiment showing *in vitro* bioactivity against trypanosomes as presented in Fig 6B. The blue lines indicate BSF parasite inhibition and the red lines show PCF parasite inhibion over the range of peptide concentrations tested. Different recPeptides are shown by varying symbols.
(TIF)

S1 Table PCR primers used in this study. (TIF)

S2 Table Detailed results and analysis of each transcriptome. (XLSX)
