# Supplementary figures and images for: Spiroplasma endosymbiont reduction of host lipid synthesis and Stomoxyn-like peptide contribute to trypanosome resistance in the tsetse fly Glossina fuscipes
Source: PLoS Pathog. 2025 Jan 31;21(1):e1012692. doi: 10.1371/journal.ppat.1012692 (PMC11819587; doi:10.1371/journal.ppat.1012692)

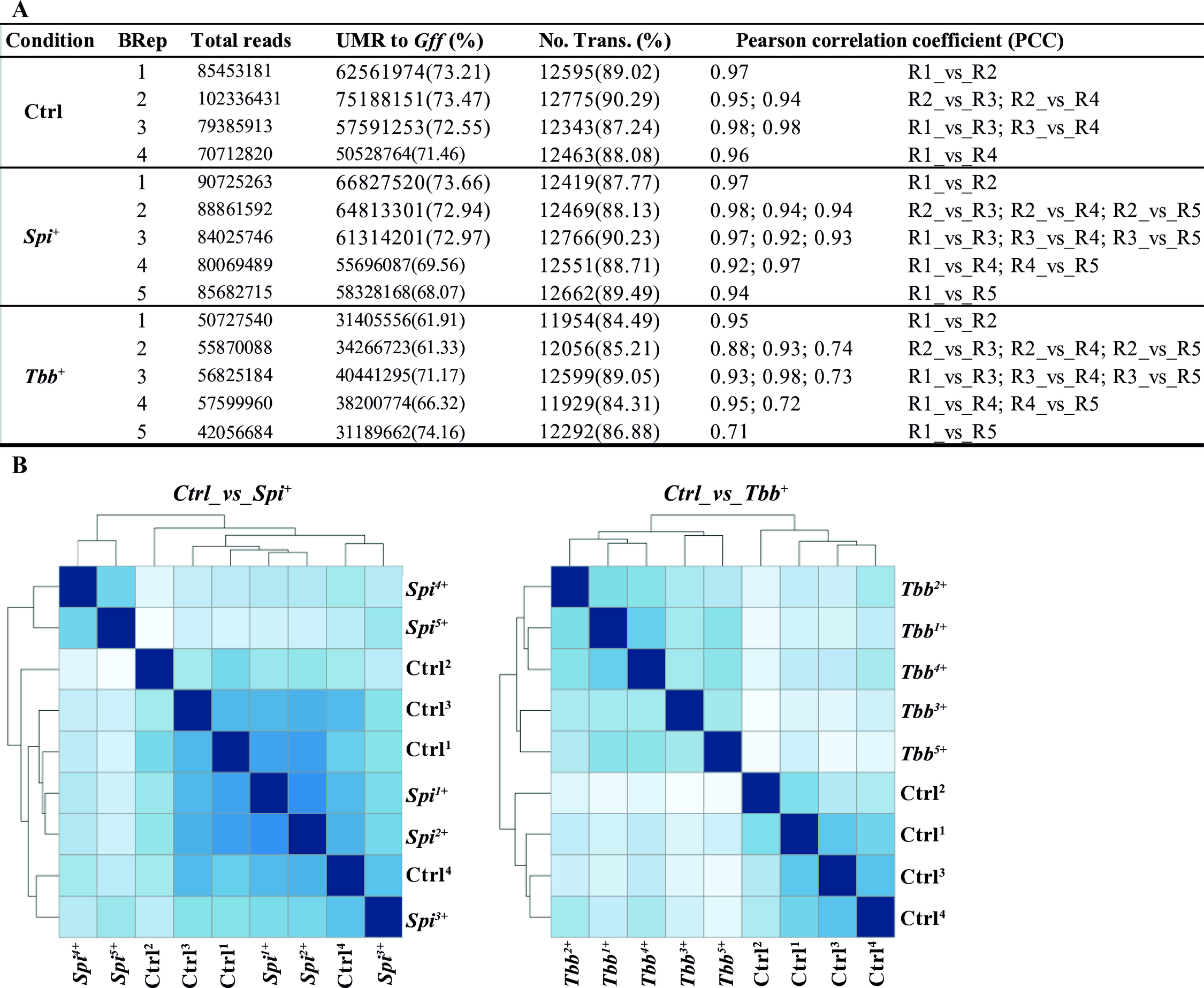

Supplement: S1 Fig — A. Table summarizes results obtained from different biological replicates across three conditions, Ctrl, Spi+ and Tbb+. Condition: Ctrl: Spiroplasma and trypanosome negative midgut; Spi+: Spiroplasma positive midgut; Tbb+: trypanosome positive midgut; BRep: Biological Replicate; UMR: Number of Uniquely Mapped Reads to the Gff genome (Gff_genome-2018_ver 63); No. Trans: Number of expressed transcripts, defined as those with normalized read coverage ≥ 10 in at least 50% of the biological replicates per condition. B. Heat map showing the Euclidean distances between biological replicates, calculated from the regularized log transformation of the data, provides insights into the similarities and differences among the conditions. (TIF) [file ppat.1012692.s001.tif]

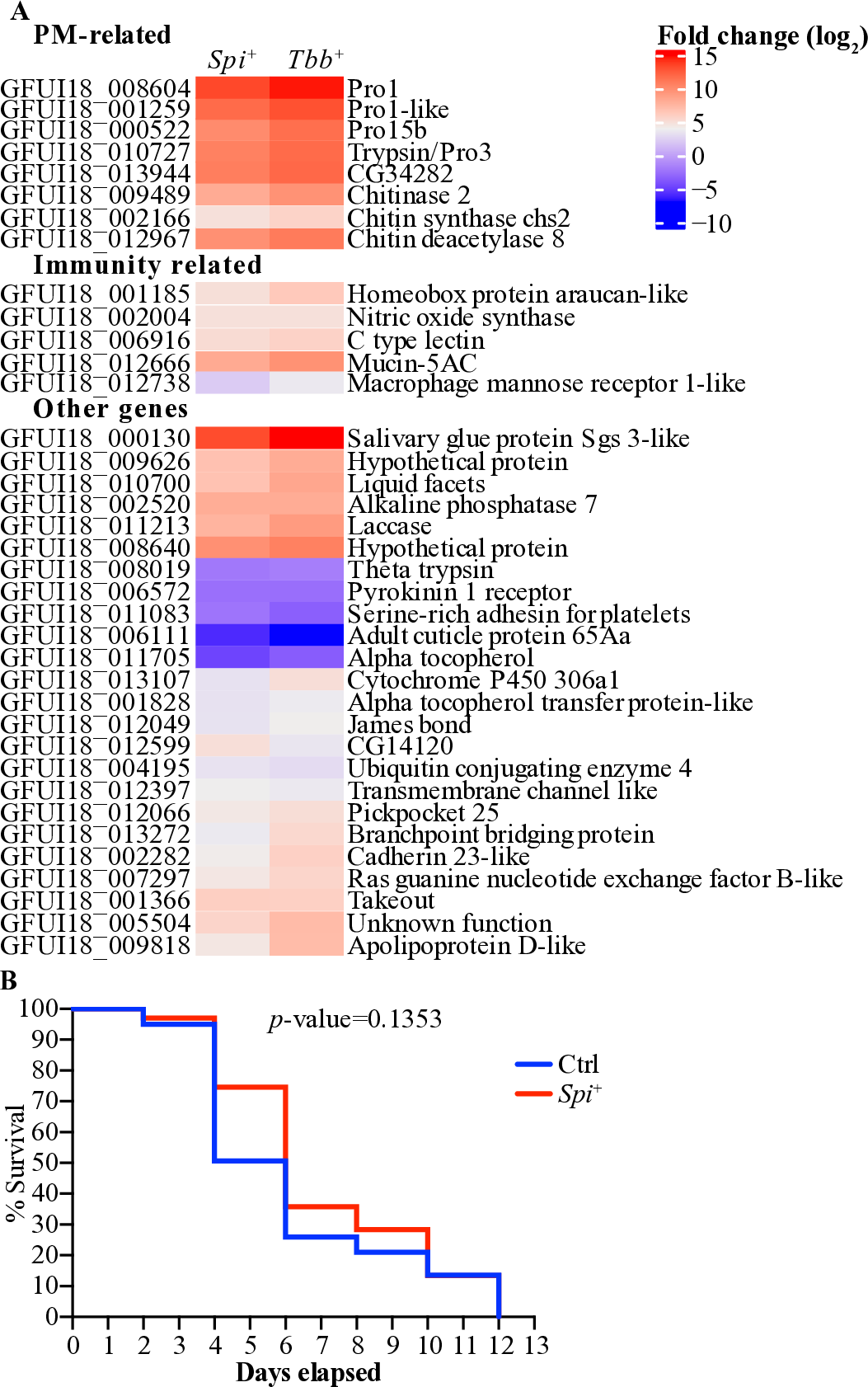

Supplement: S2 Fig — The Heat map denotes the fold changes of differentially expressed (DE) genes that are shared between the Spi+ and Tbb+ states, according to their putative functions. Fold-change values are expressed as a fraction of the average normalized gene expression levels from age-matched Spi+ or Tbb+ relative to the control Ctrl. The heat maps (dendrograms) were generated using Euclidean distance calculation combined with ward.D clustering methods within the R-package software. The clusters were manually separated into two categories: PM and Immunity Functions. B. Effect of Spiroplasma infection on Peritrophic Matrix integrity. The survival of flies was monitored every 48 h following a per os treatment of teneral adult flies with Serratia marcescens, administered 72 h post-eclosion. At time of death, the Spiroplasma infection status of each fly was evaluated using our diagnostic assay. The Kaplan-Meyer survival curves illustrate the fly survival over time for Spiroplasma-uninfected flies (blue) and Spiroplasma-infected flies (red). This experiment was conducted twice, with no significant differences observed between the two experiments. (TIF) [file ppat.1012692.s002.tif]

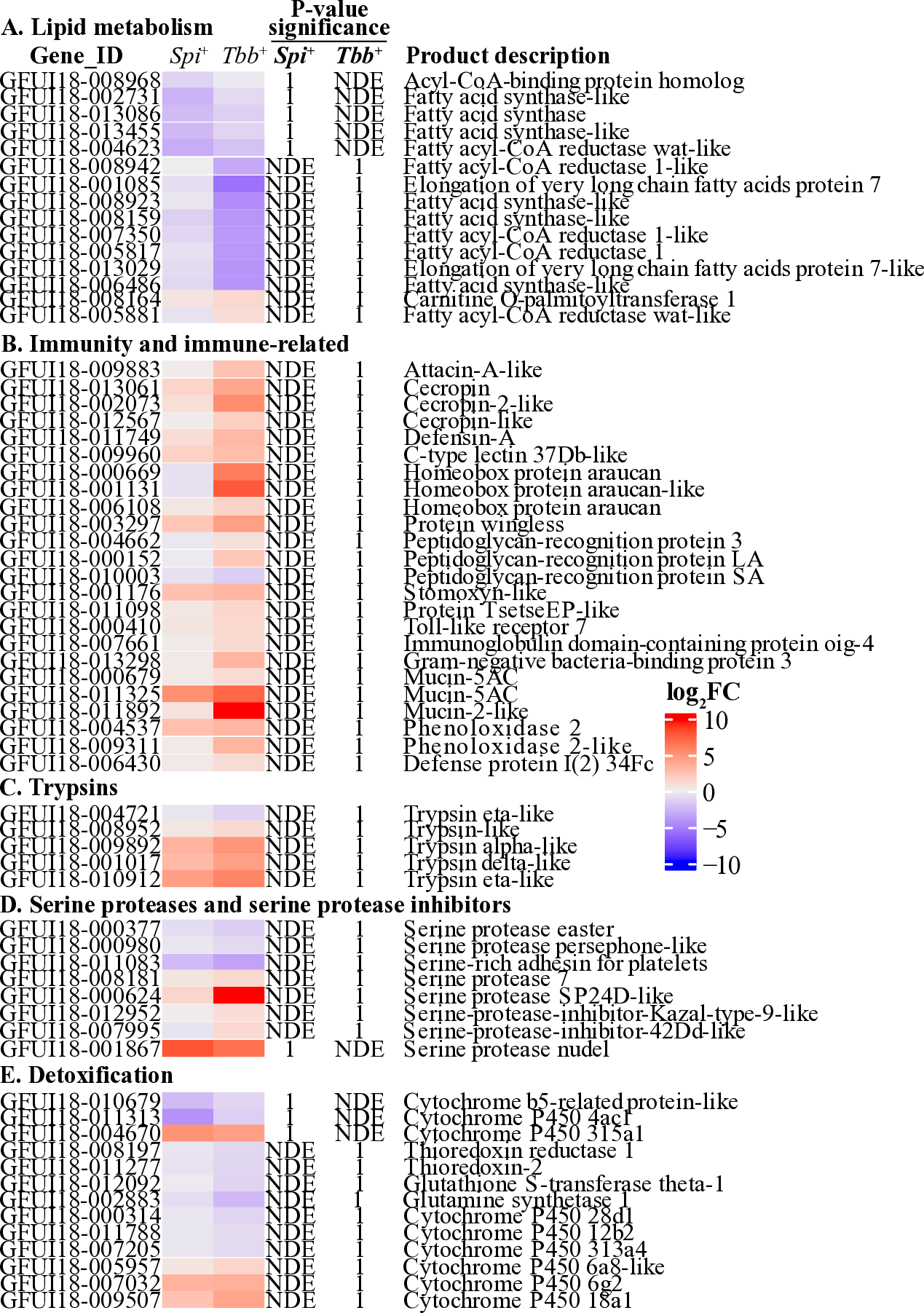

Supplement: S3 Fig — The heatmaps depict the fold changes of unique DE transcripts across various functional categories, comparing infected transcriptomes and the uninfected control. Spi+: Spiroplasma infected, Tbb+: trypanosome infected; FC: fold change indicate the degress of change in expression levels relative to uninfected controls; 1: differentially expressed transcripts; NDE: transcripts that are not differentially expressed. (TIF) [file ppat.1012692.s003.tif]

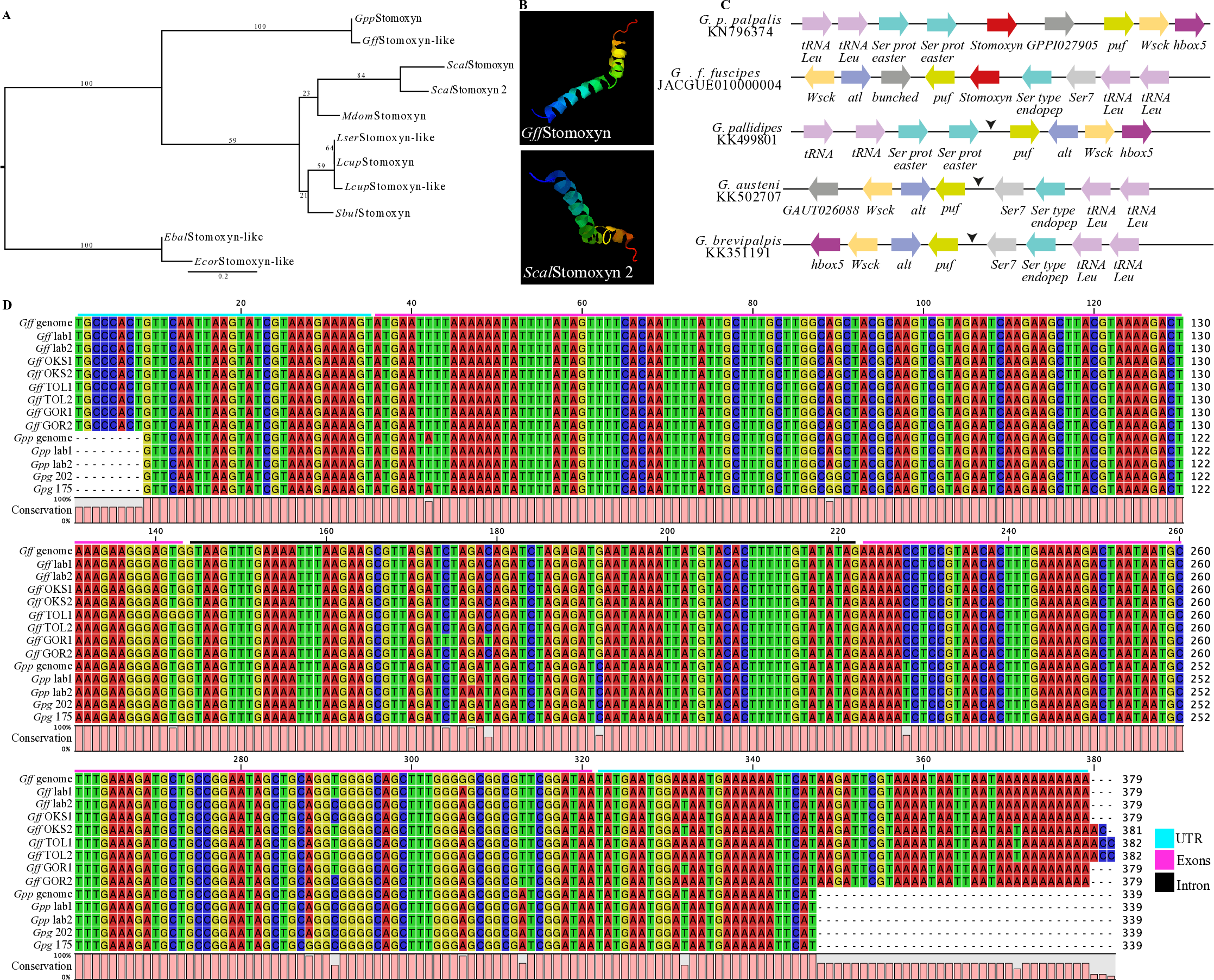

Supplement: S4 Fig — A. Phylogenetic tree of mature Stomoxyn sequences from nine different Diptera species based the Maximum likelihood (ML) model. Sequences used in this analysis were obtained from VectorBase for S. calcitrans (ScalStomoxyn; SCAU016937 and ScalStomoxyn 2; SCAU016907), Gff (GffStomoxyn-like; GFUI18_001176), Gpp (GppStomoxyn; GPPI027903) and M. domestica (MdomStomoxyn-like; MDOA008330), and from NCBI database for L. cuprina (LcupStomoxyn; KAI8119624.1 and LcupStomoxyn-like; XP_023308701.2), S. bullata (SbulStomoxyn; DOY81_004902), L. sericata (Lserstomoxyn-like; XP_037825072.1), Episyrphus balteatus (EbalStomoxyn-like; XP_055851874.1) and Eupeodes corollae (EcorStomoxyn-like; XP_055904620.1). The analysis involved 11 amino acid sequences and 1000 bootstrap replications. B. The tertiary structure of the mature GffStomoxyn and ScalStomoxyn 2 peptide was predicted by I-TASSER. C. Genomic content surrounding the Stomoxyn-like gene (GFUI18_001176) focusing on the supercontig JACGUE010000004 of the Gff genome assembly (version 63, Vectorbase). The supercontigs available from the other Glossina WGS data were also compared. Genes exhibiting synteny among different tsetse species are indicated by arrows with the same color genes that do not shown synteny are presented in gray. The black arrow marks the expected location of the Stomoxyn gene in G. pallidipes, G. austeni and G. brevipalpis. Gene size and spacings are not drawn to scale. Based on the current assembly of the region, only one Stomoxyn gene is present in Gff and Gpp, while it is absent in other Glossina species. D. Multiple sequence alignment of the Stomoxyn locus from Gff, Gpp and Gpg. The alignment includes genomic PCR product sequences of the Stomoxyn locus from flies obtained from laboratory and field populations, confirming the presence and conservation of this locus in the species from Palpalis subgroup. The primers for the PCR amplification were designed to span the entire coding region of the Stomoxyn pre-pro-m [file ppat.1012692.s004.tif]

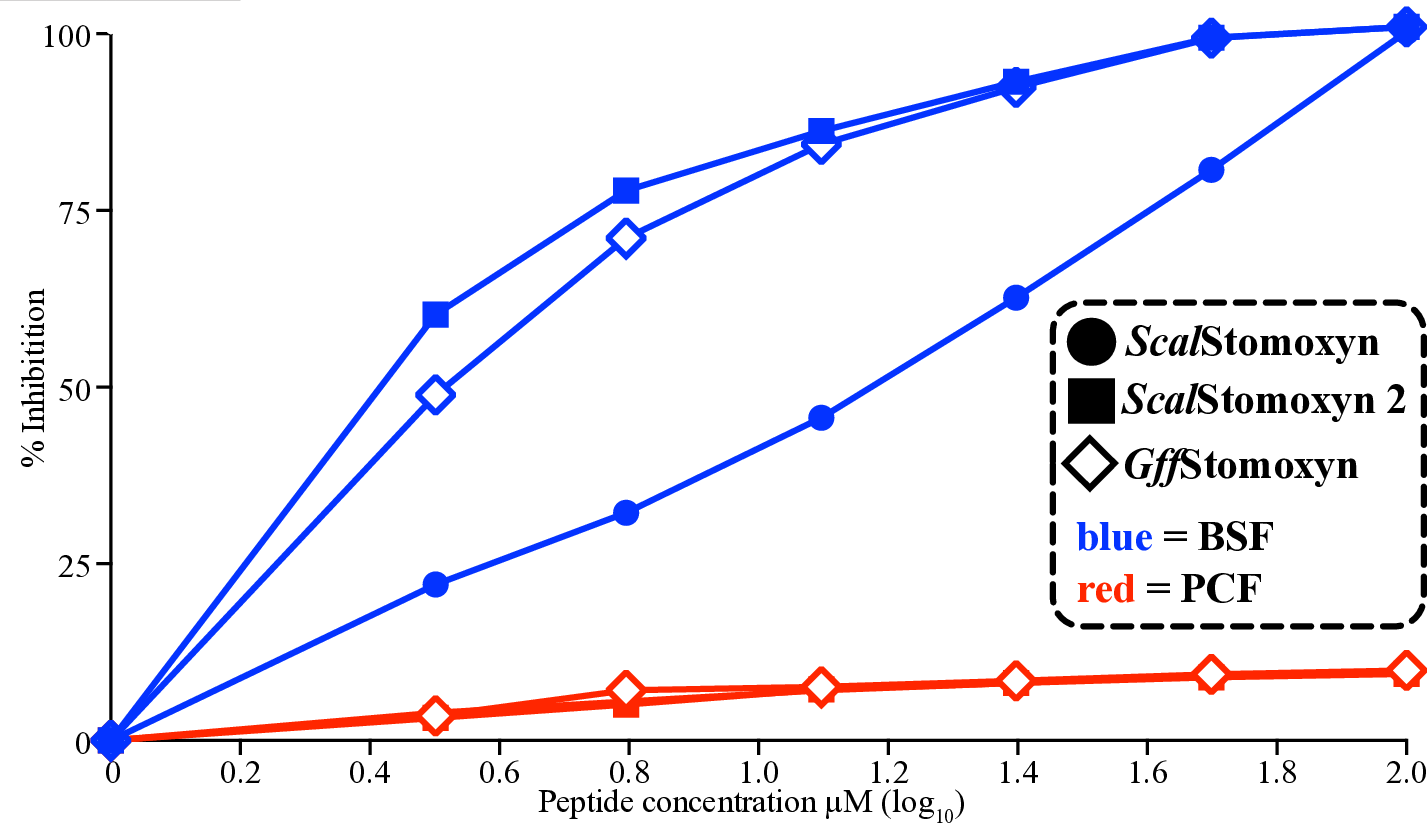

Supplement: S5 Fig — A replicate experiment showing in vitro bioactivity against trypanosomes as presented in Fig 6B. The blue lines indicate BSF parasite inhibition and the red lines show PCF parasite inhibion over the range of peptide concentrations tested. Different recPeptides are shown by varying symbols. (TIF) [file ppat.1012692.s005.tif]

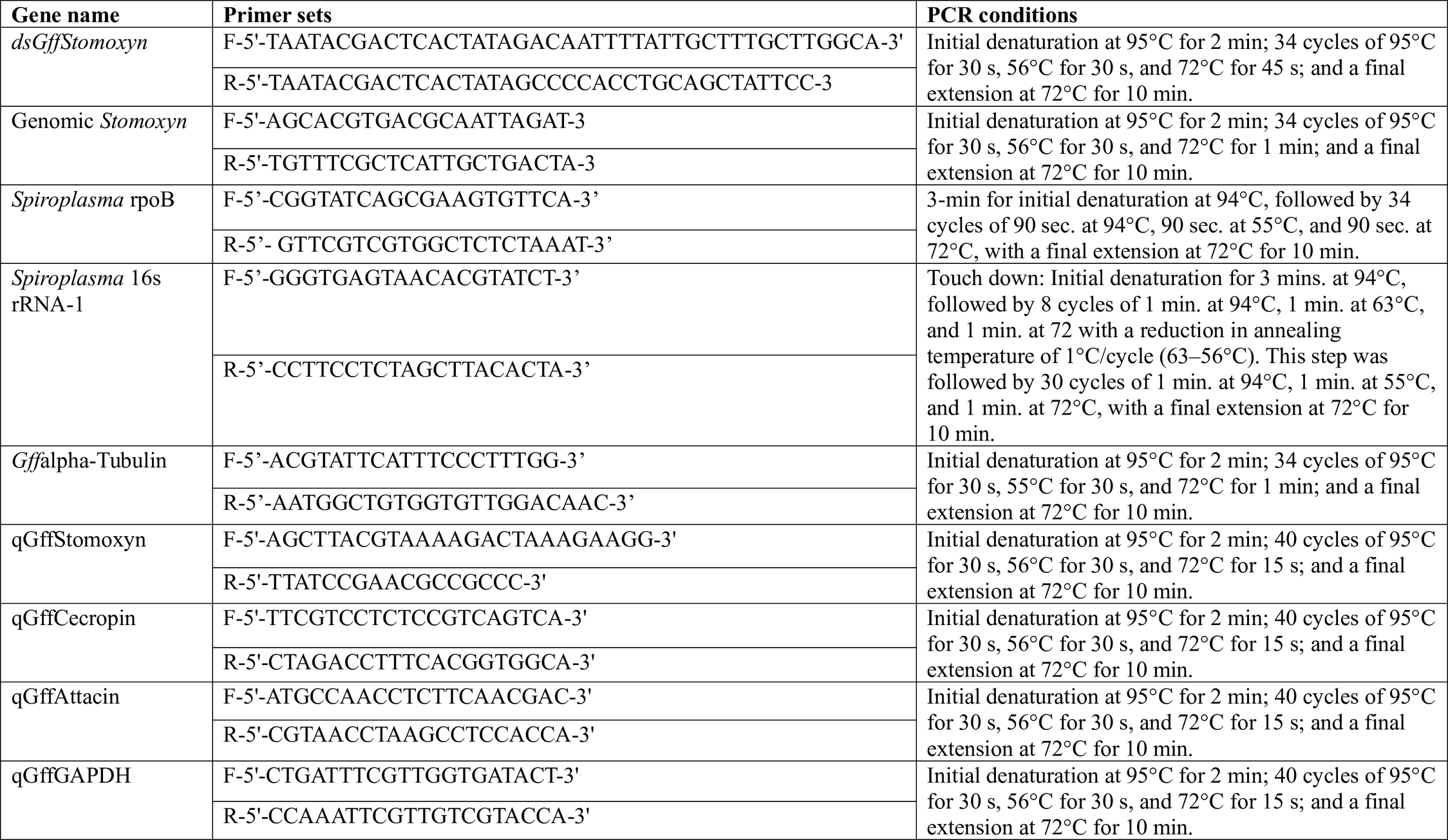

Supplement: S1 Table — (TIF) [file ppat.1012692.s006.tif]
